# Supplementary material for: Super enhancer regulation of cytokine-induced chemokine production in alcoholic hepatitis
Source: Nat Commun. 2021 Jul 27;12:4560. doi: 10.1038/s41467-021-24843-w (PMC8316465; doi:10.1038/s41467-021-24843-w)
Supplement: Supplementary file 1 — Supplementary Information [file 41467_2021_24843_MOESM1_ESM.pdf]

Supplementary Figure 1 | Clinical Characteristics of AH patients

	Control Group (n=4)	Alcoholic Hepatitis Group (n=6)
Age (years)	60 (58-71)	56 (32-60)
MELD score	6	30 (25-35)
AST (IU/L)	30 (29-39)	114 (73-199)
ALT (IU/L)	29 (22-34)	37 (25-61)
Creatinine (mg/dL)	0.9 (0.7-1.2)	1.1 (0.75-2.5)
Total bilirubin (mg/dL)	0.6 (0.3-0.8)	21.3 (12.3-29.5)
Leukocytes ($x10^3$)	6.2 (5-8.7)	14.8 (8.4-23.3)
Platelets ($x10^3$)	153 (135-399)	106 (87-164)
Prothrombin time (%)	98 (86-100)	28.5 (23.5-42.8)

Supplementary Figure 2

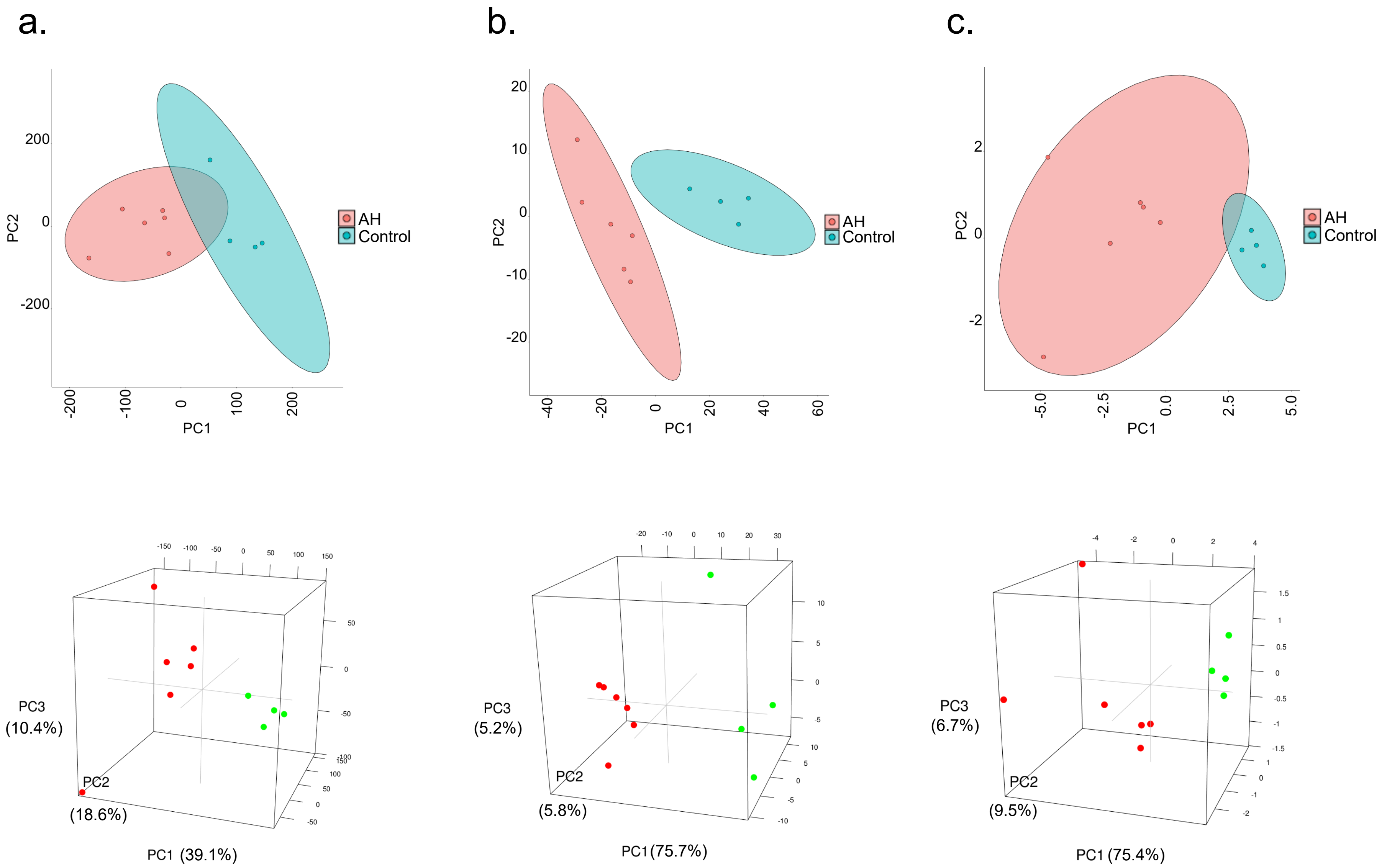

Supplementary Figure 3

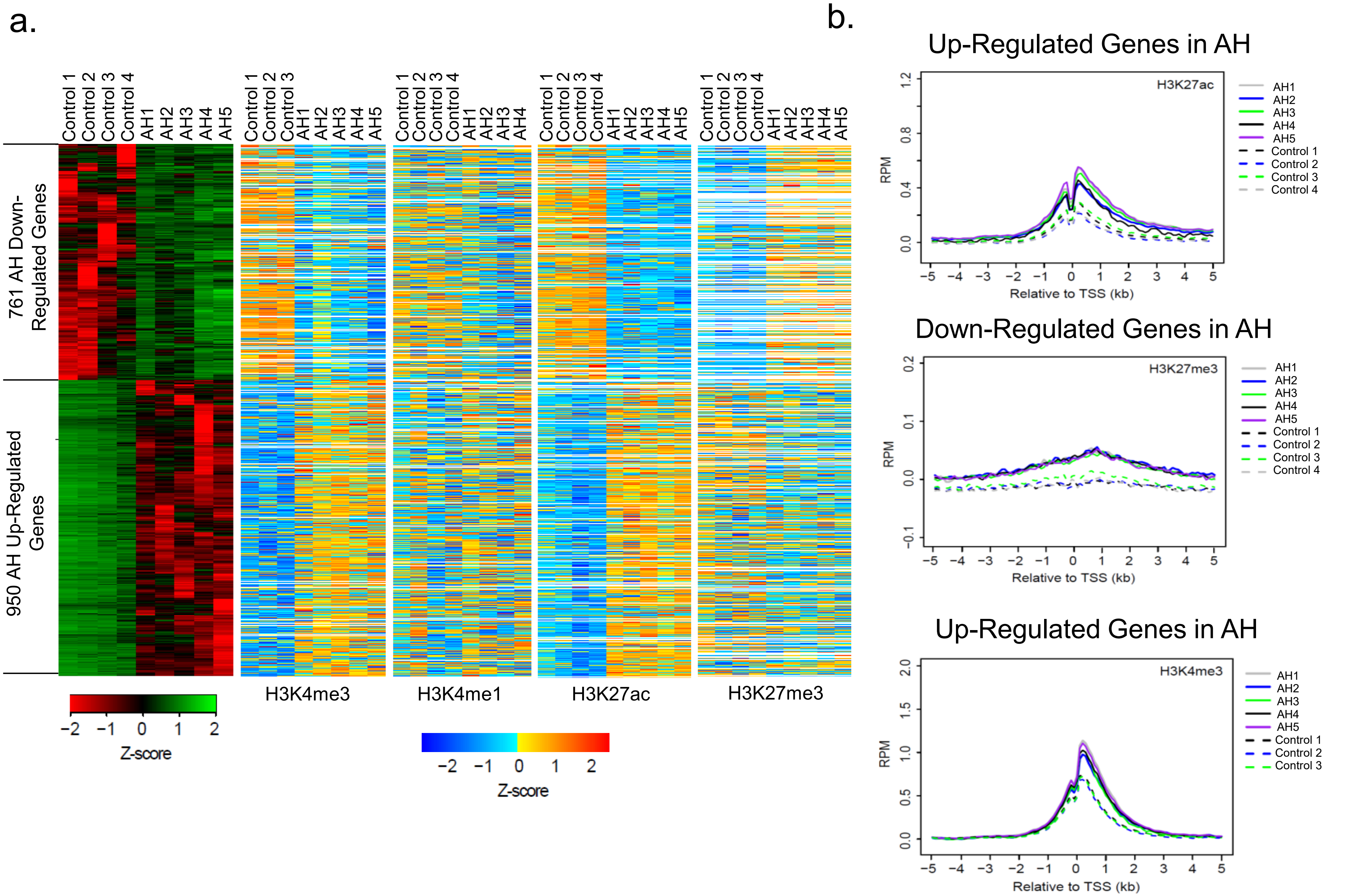

Supplementary Figure 4

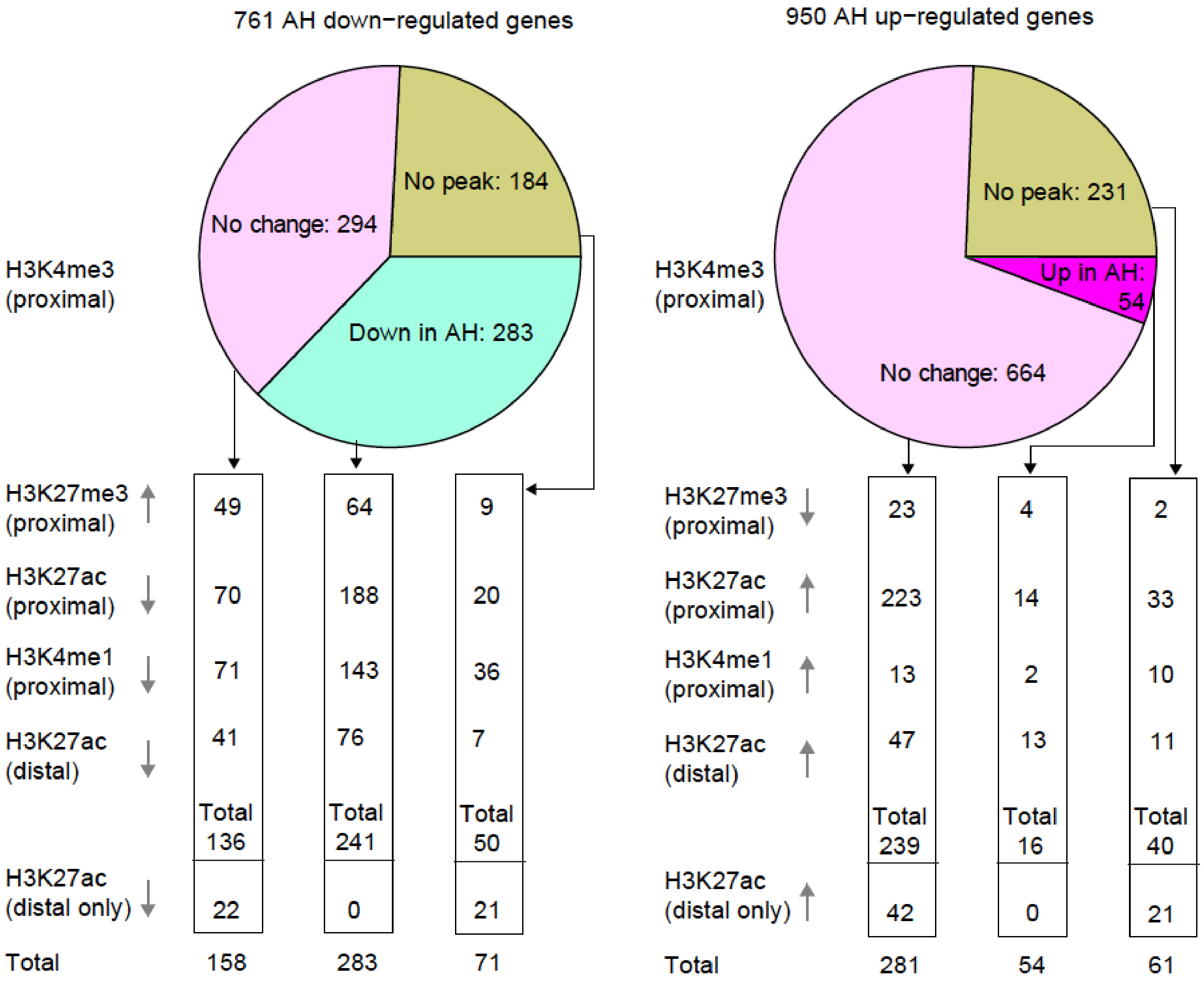

Supplementary Figure 5

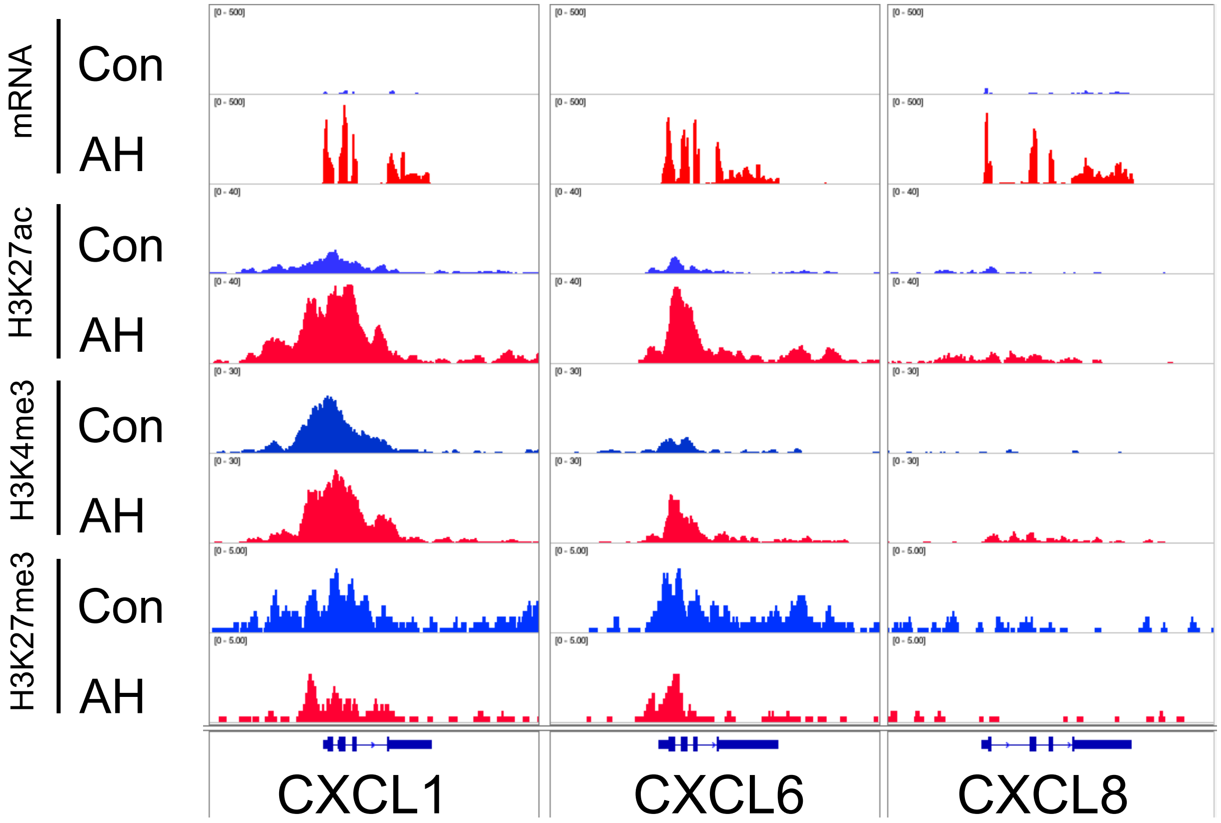

Red: AH patients
Blue: Healthy Controls

Supplementary Figure 6

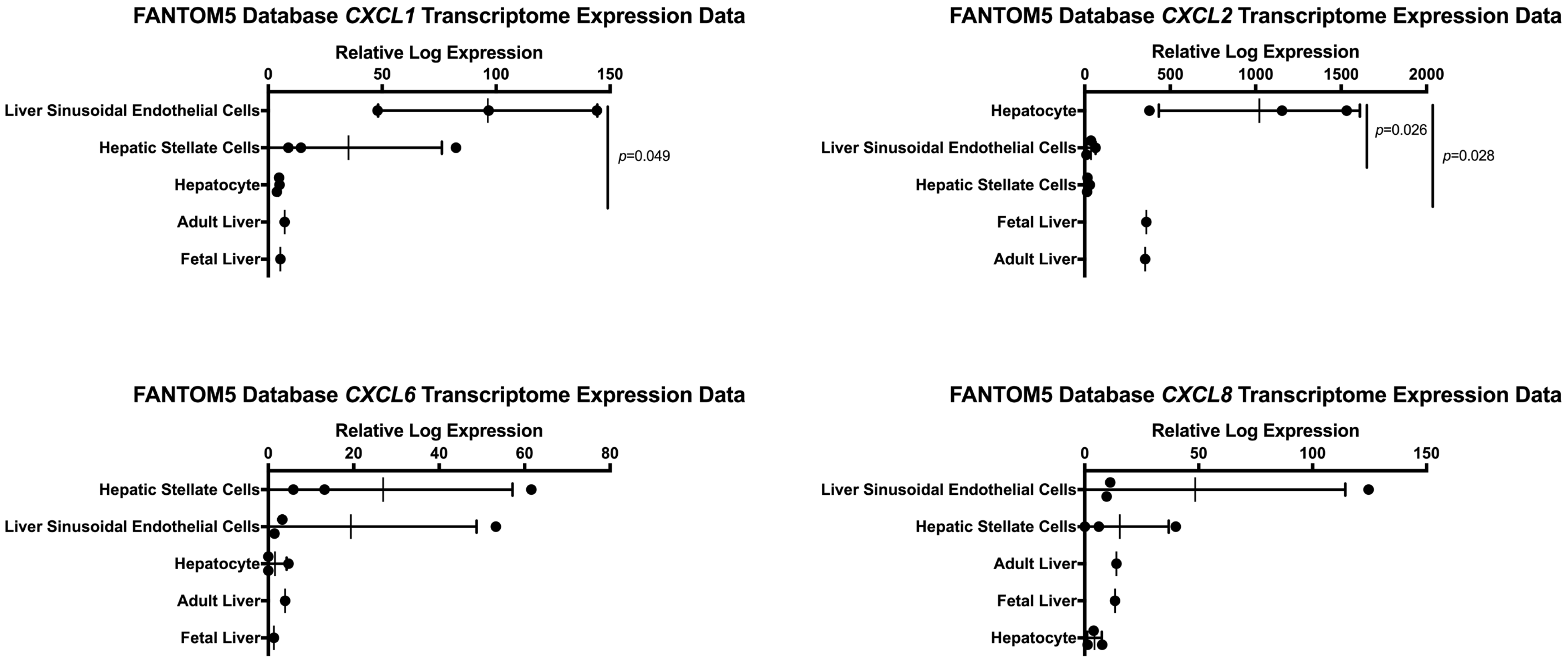

Supplementary Figure 7

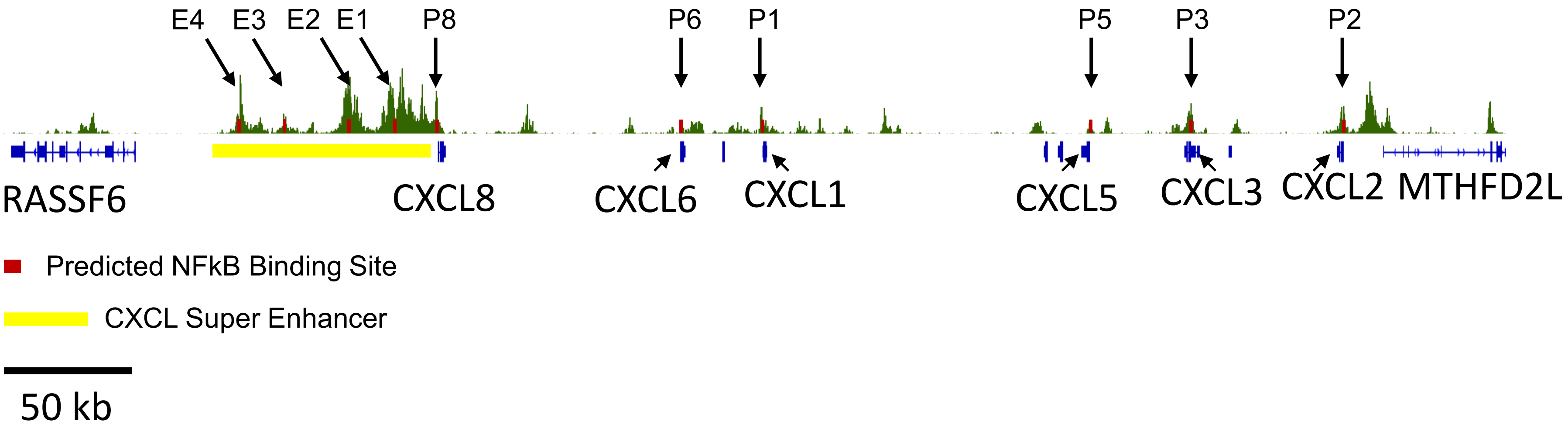

Supplementary Figure 8

a.

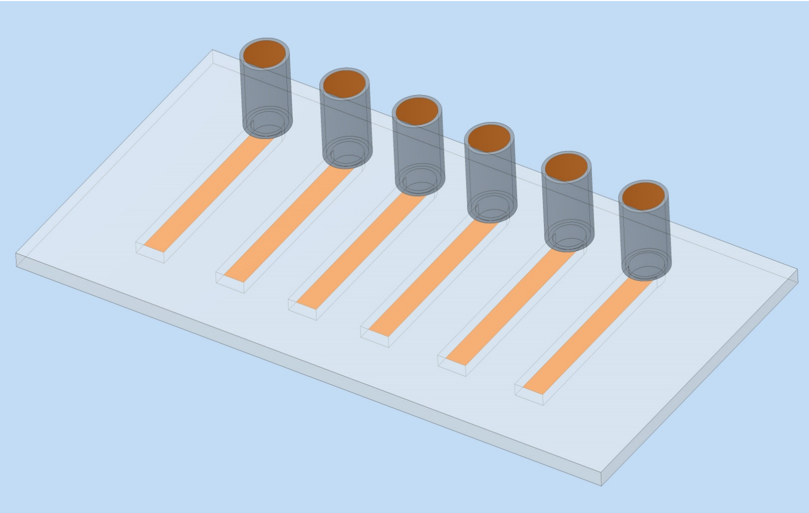

c.

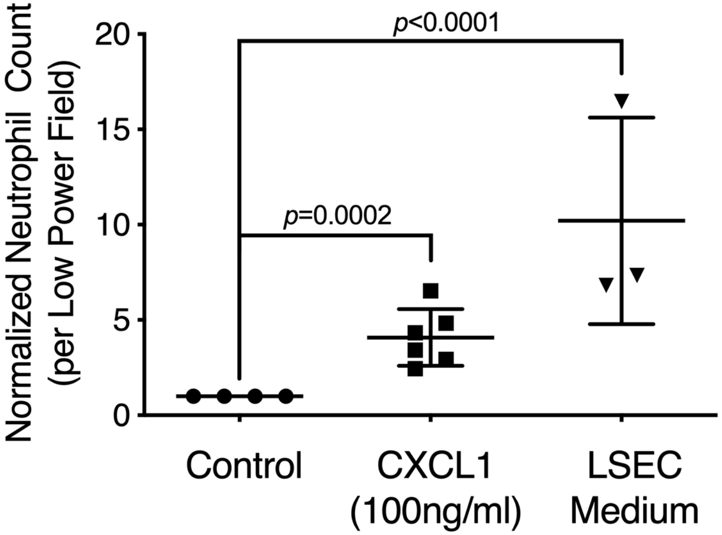

b.

Control Condition

100 ng/ml CXCL1

LSEC Medium

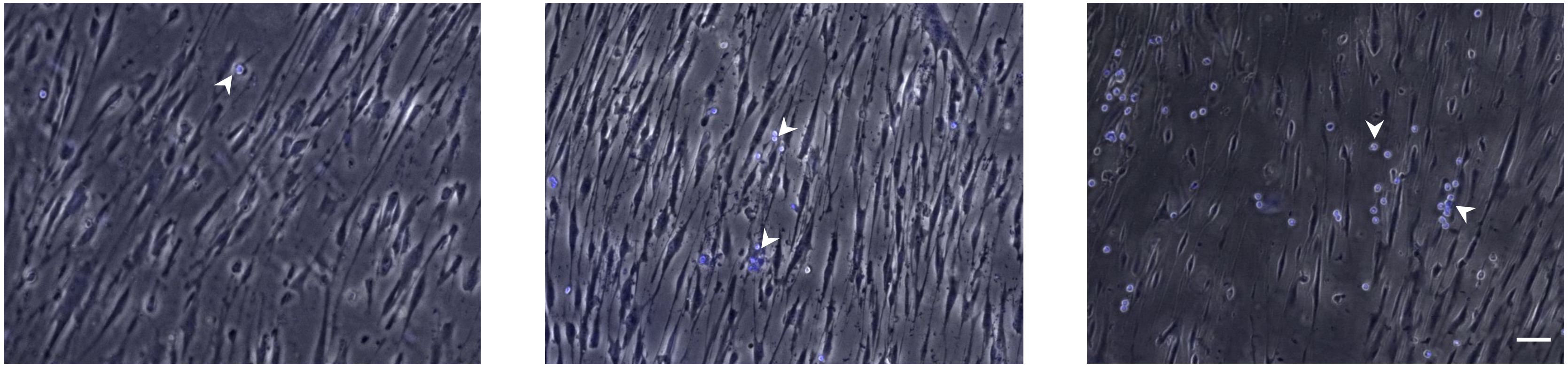

Supplementary Figure 9

a.

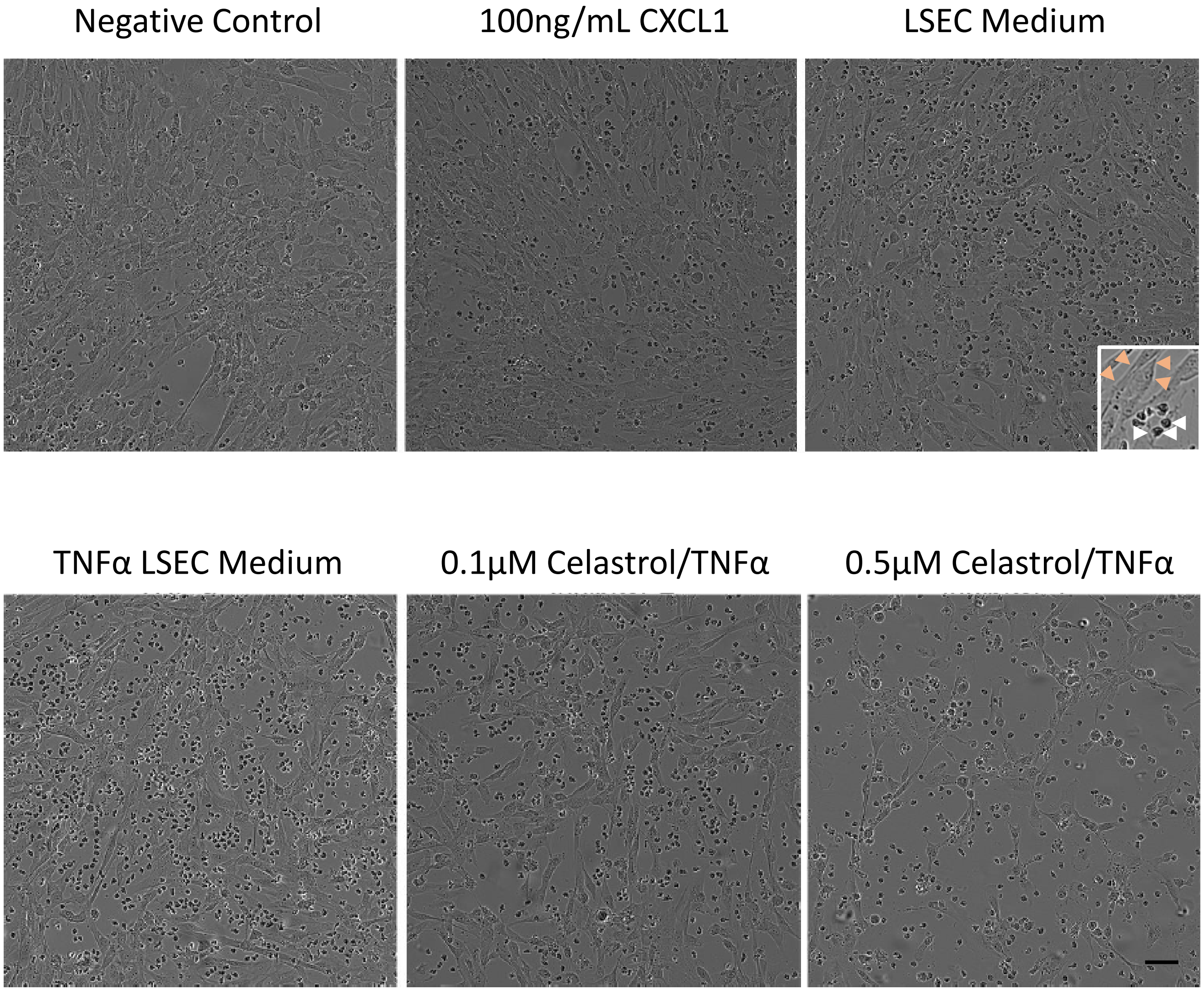

b.

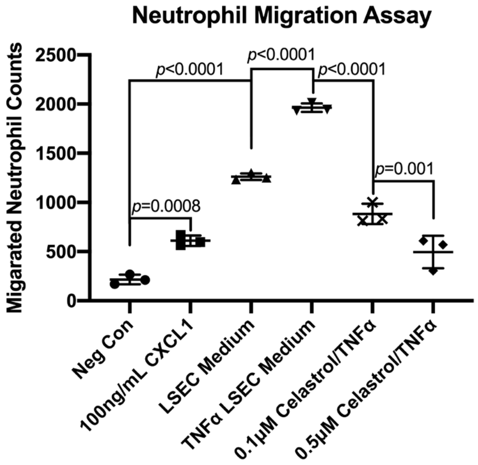

Supplementary Figure 10

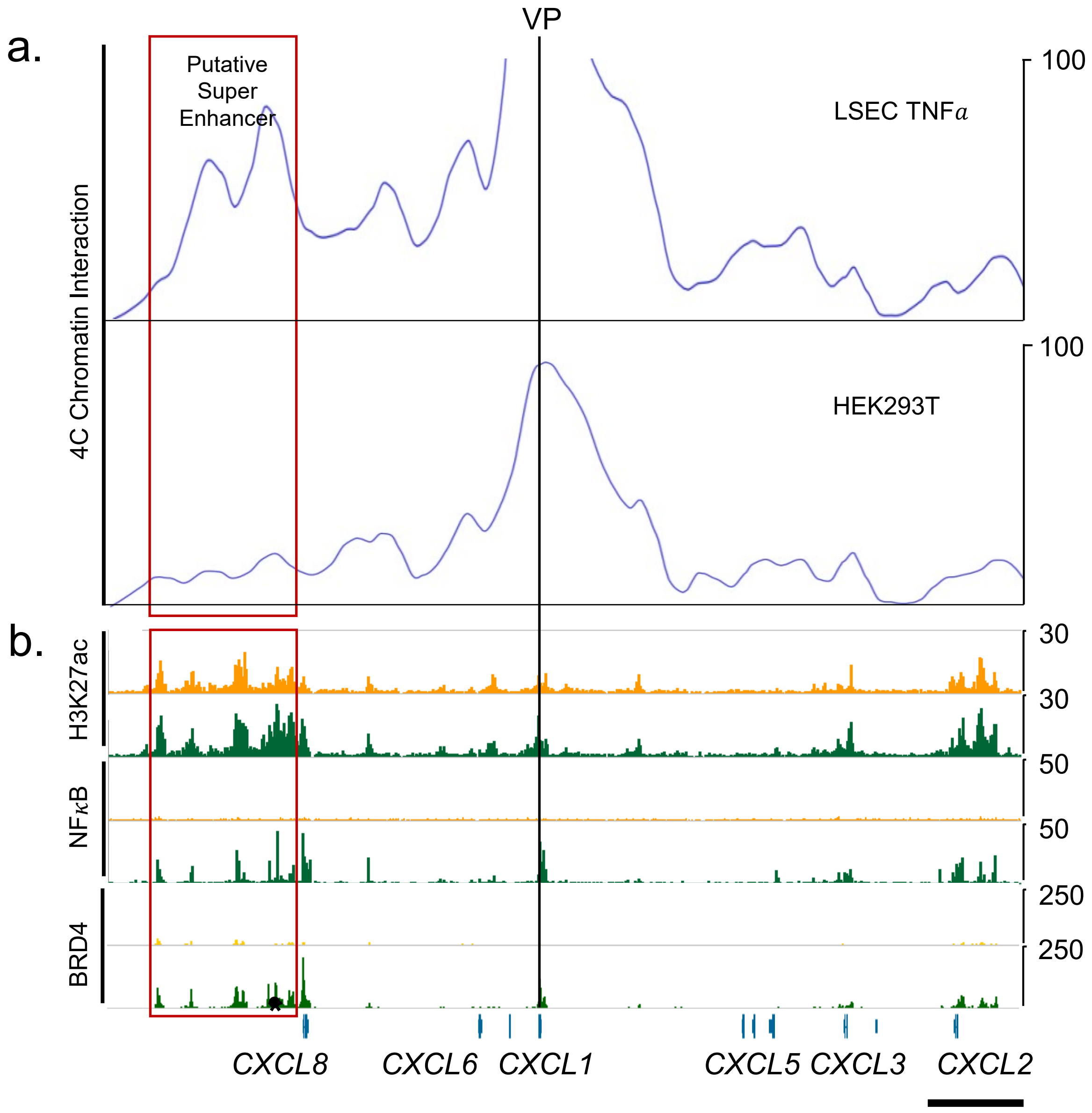

Supplementary Figure 11

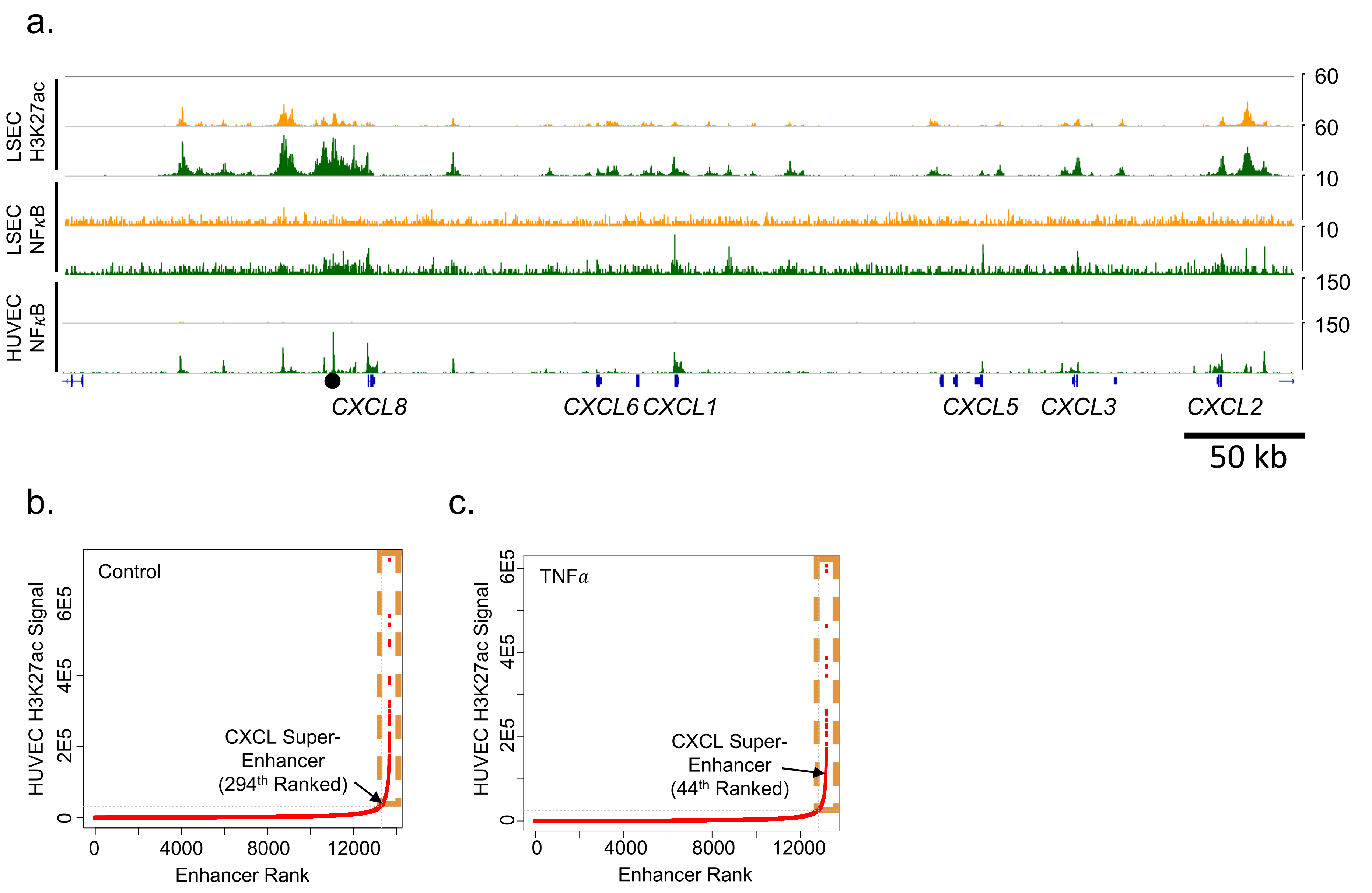

Supplementary Figure 12

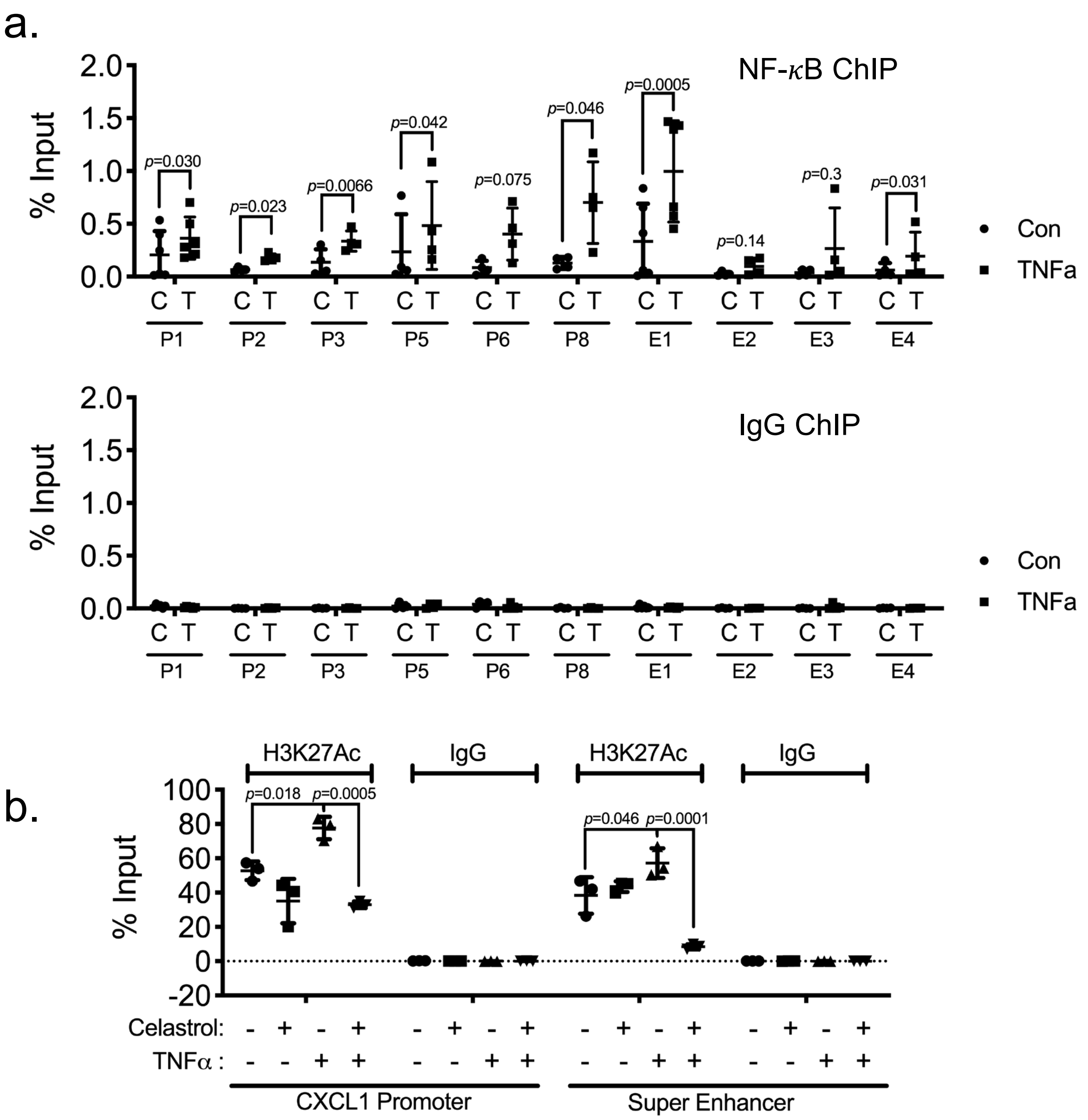

Supplementary Figure 13

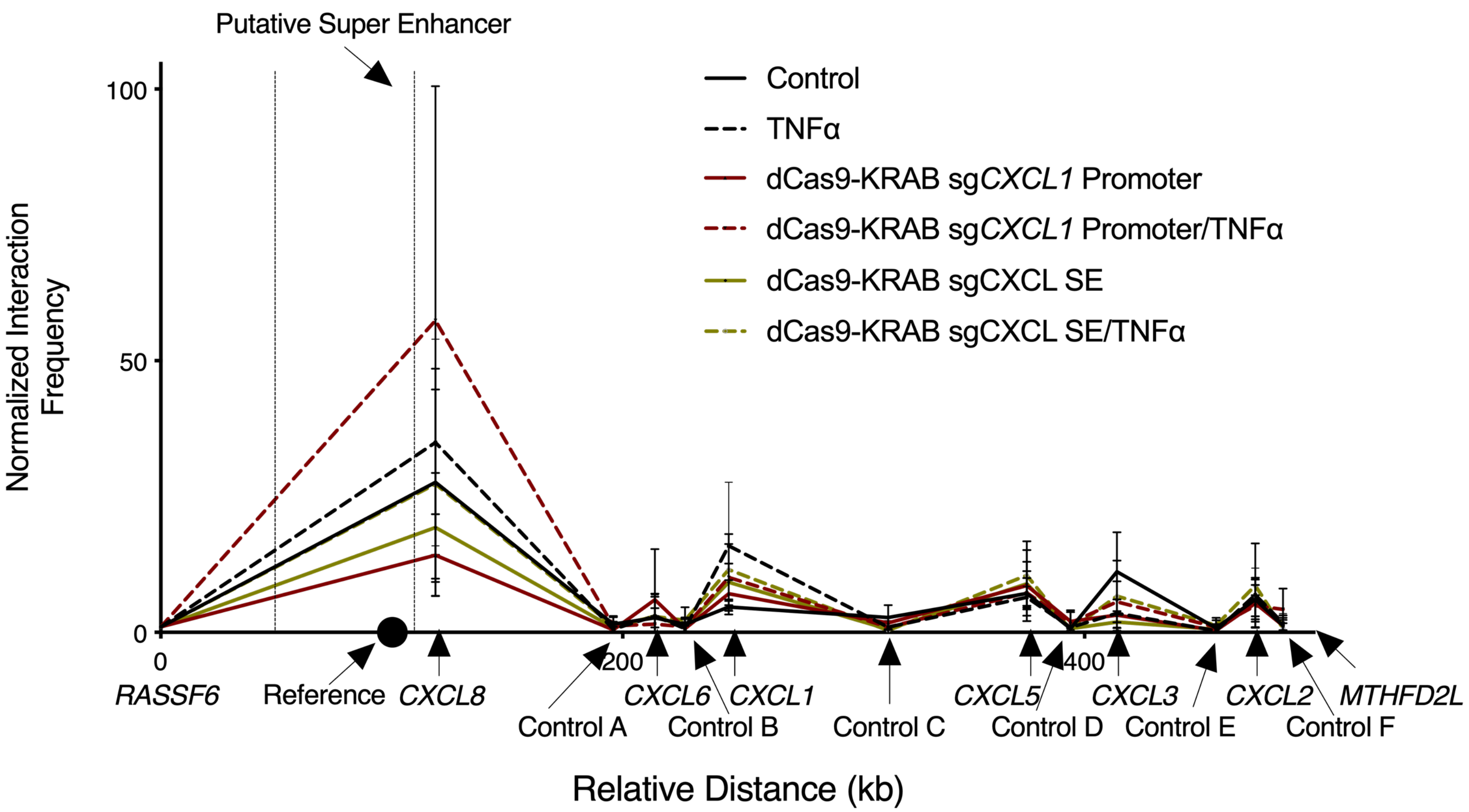

Supplementary Figure 14

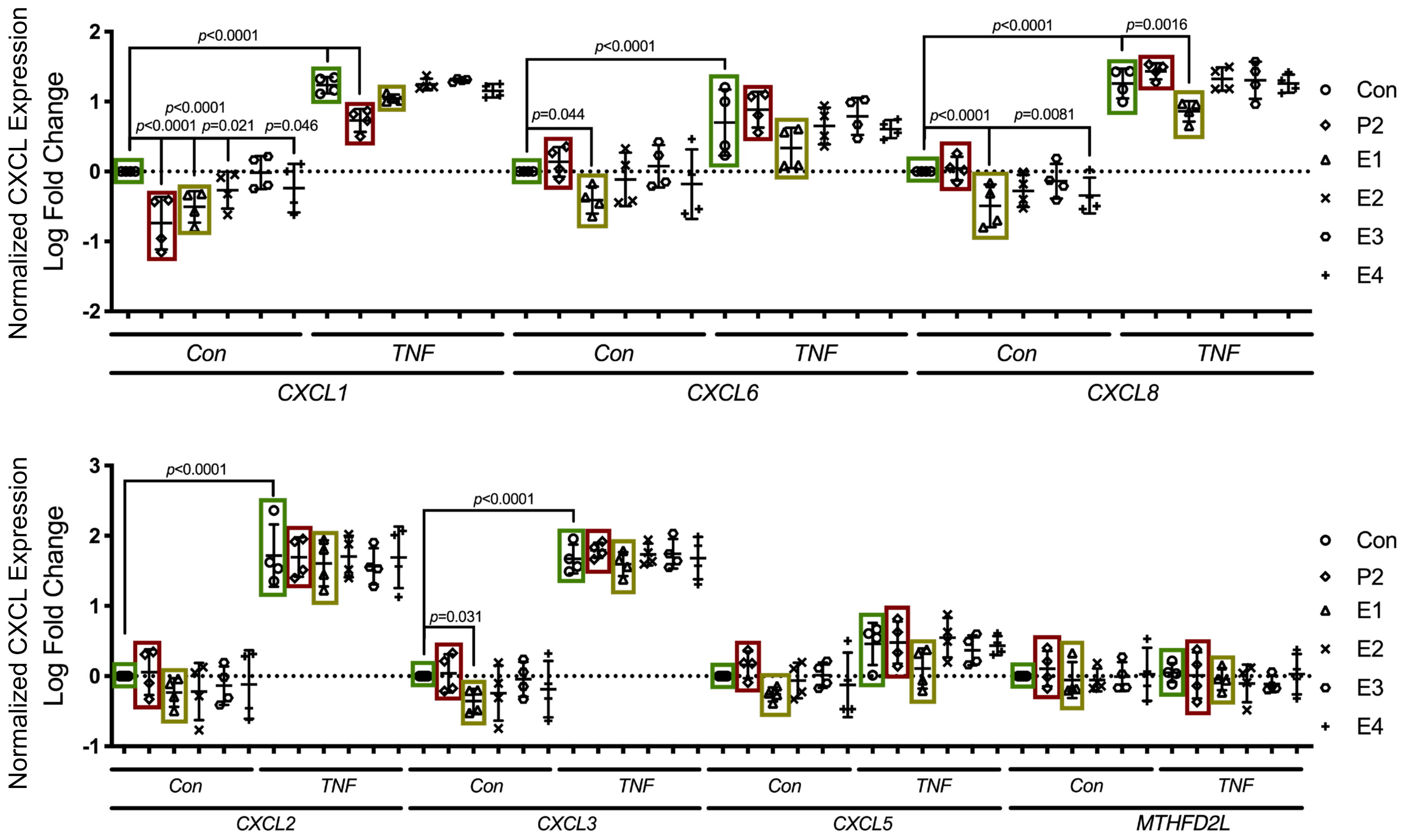

Supplementary Figure 15

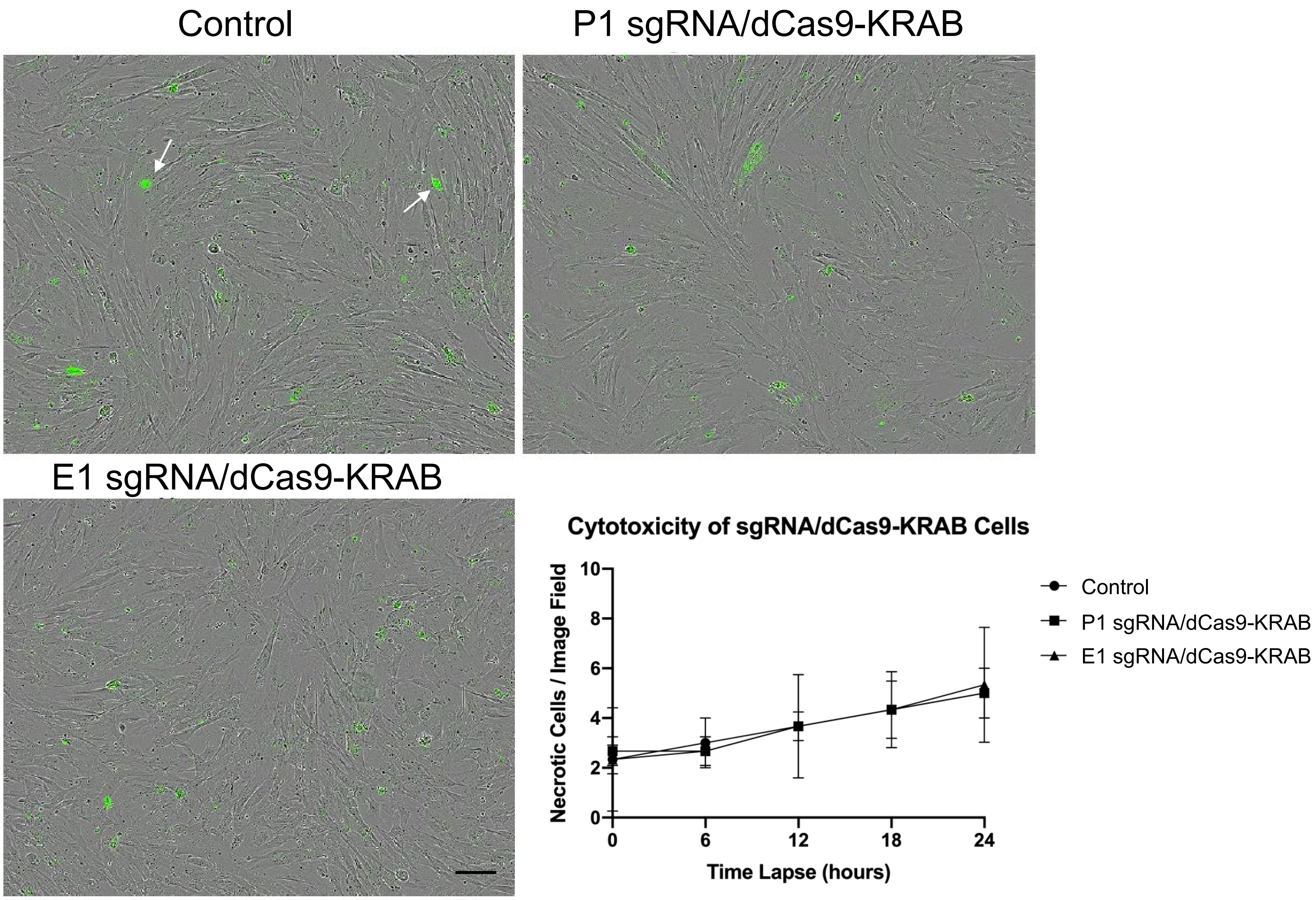

Supplementary Figure 16

a. sgRNA targeting CXCL1 SE/ With dCas9-KRAB

b. sgRNA targeting CXCL1 Promoter/ With dCas9-KRAB

c. sgRNA targeting CXCL1 SE/ No KRAB

d. sgRNA targeting CXCL1 Promoter/ No KRAB

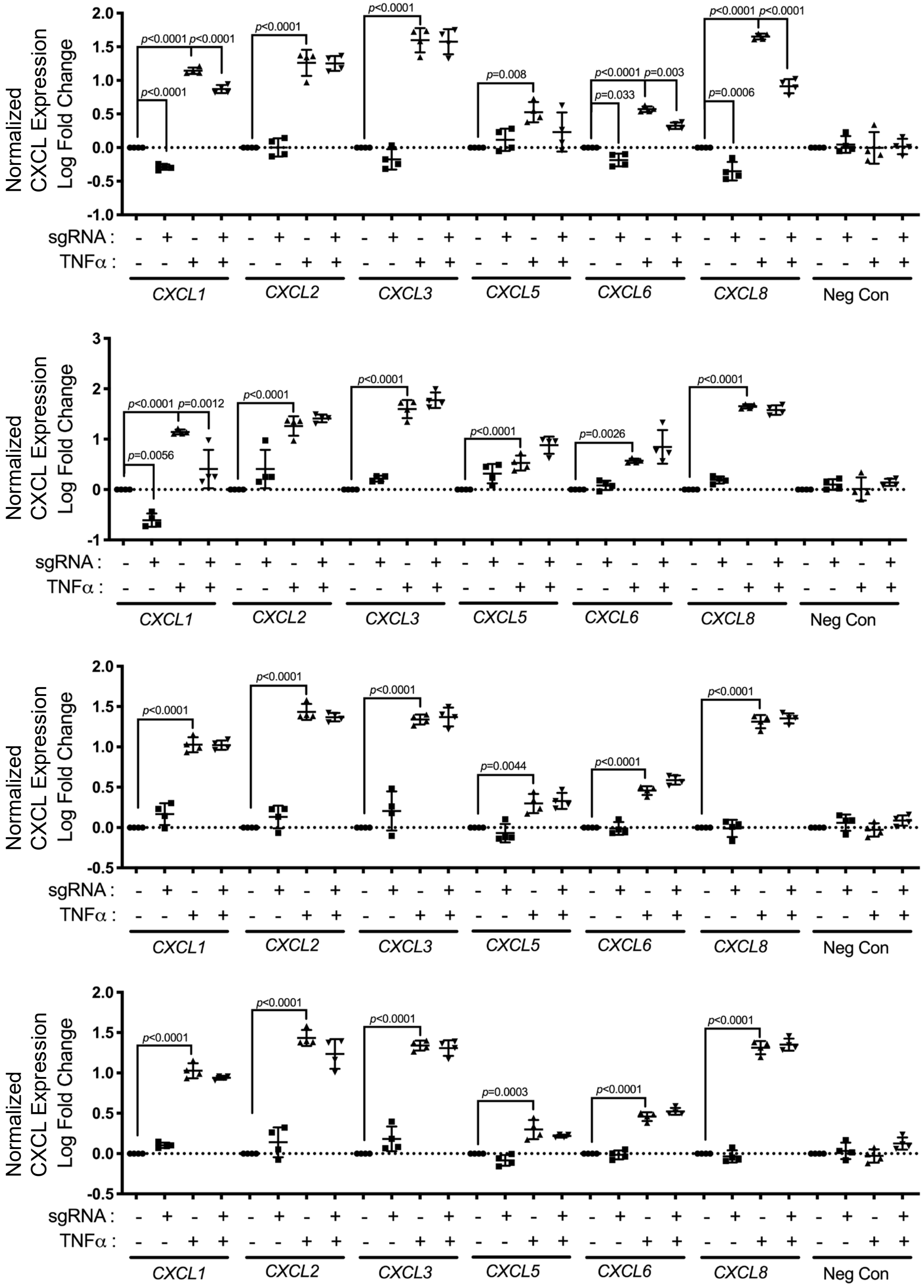

Supplementary Figure 17

a. sgRNA targeting CXCL1 SE/
With dCas9-FLAG

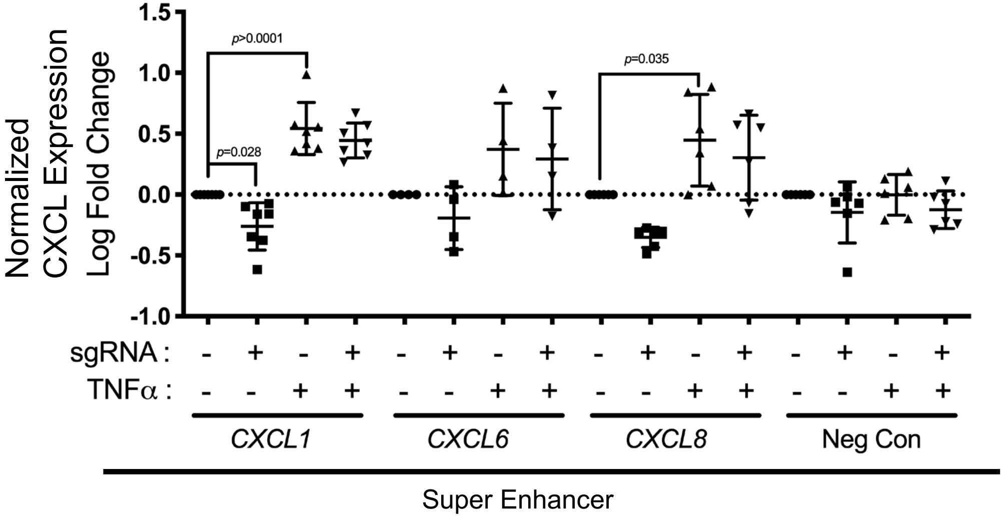

b. sgRNA targeting CXCL1 Promoter/
With dCas9-FLAG

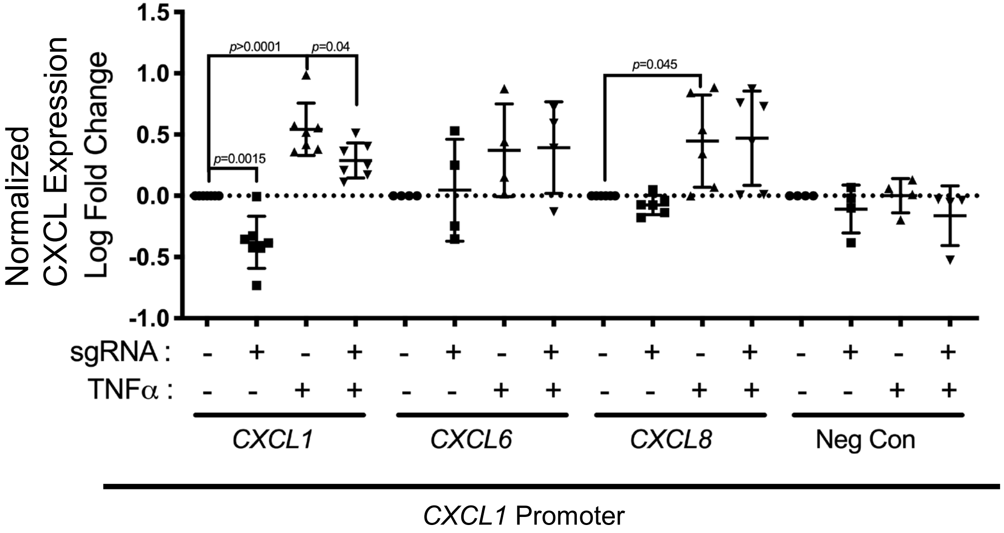

Supplementary Figure 18

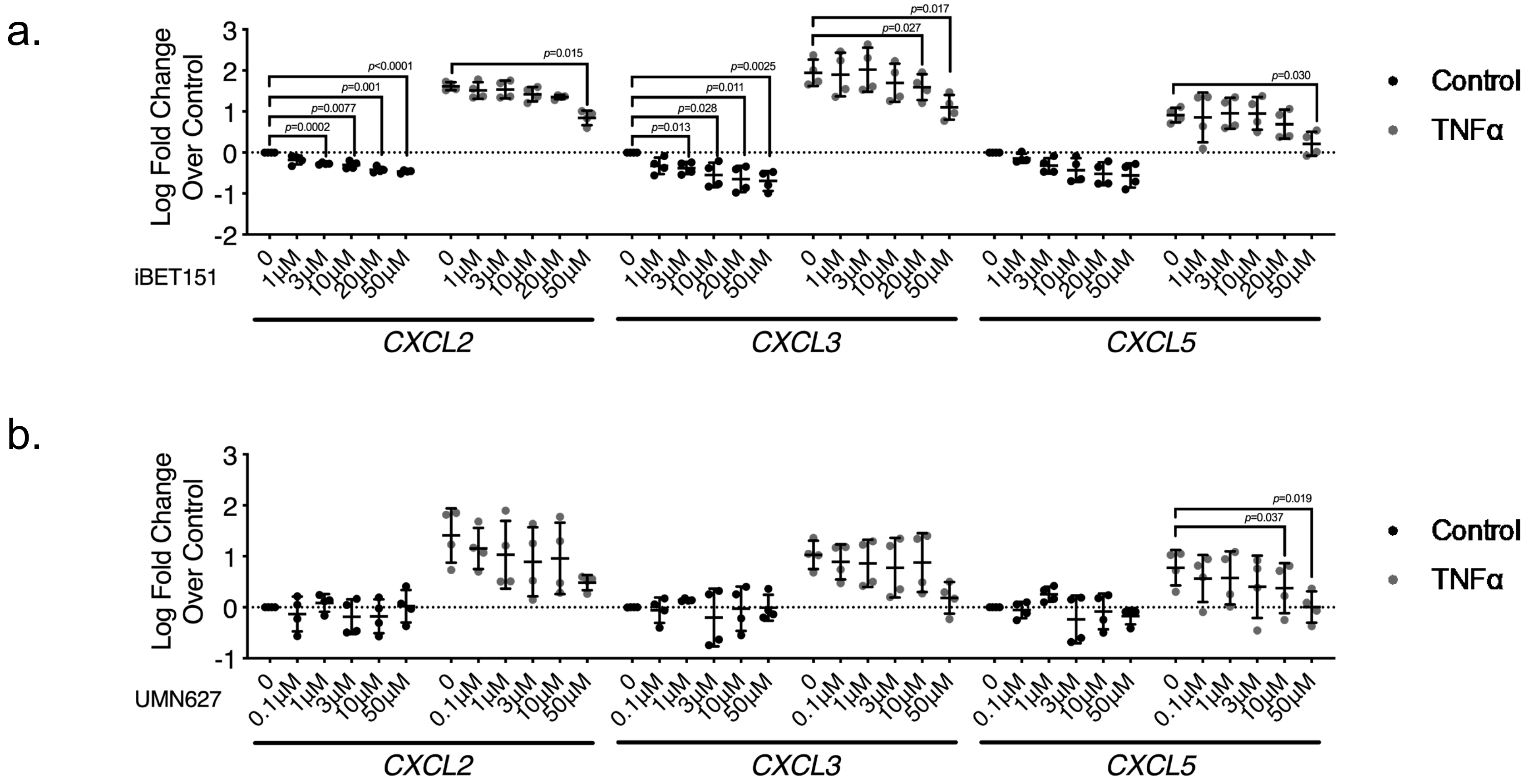

Supplementary Figure 19

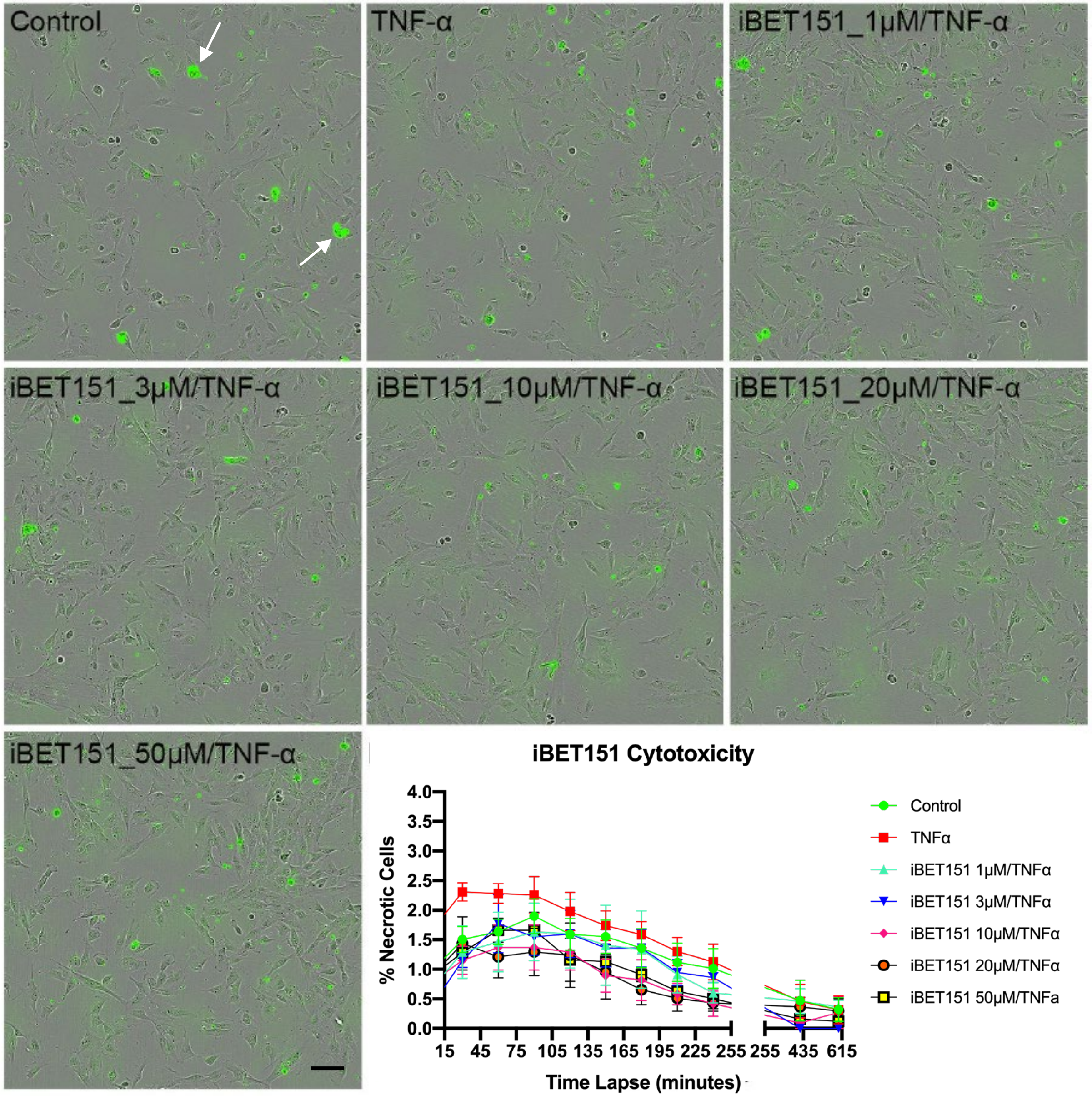

Supplementary Figure 20

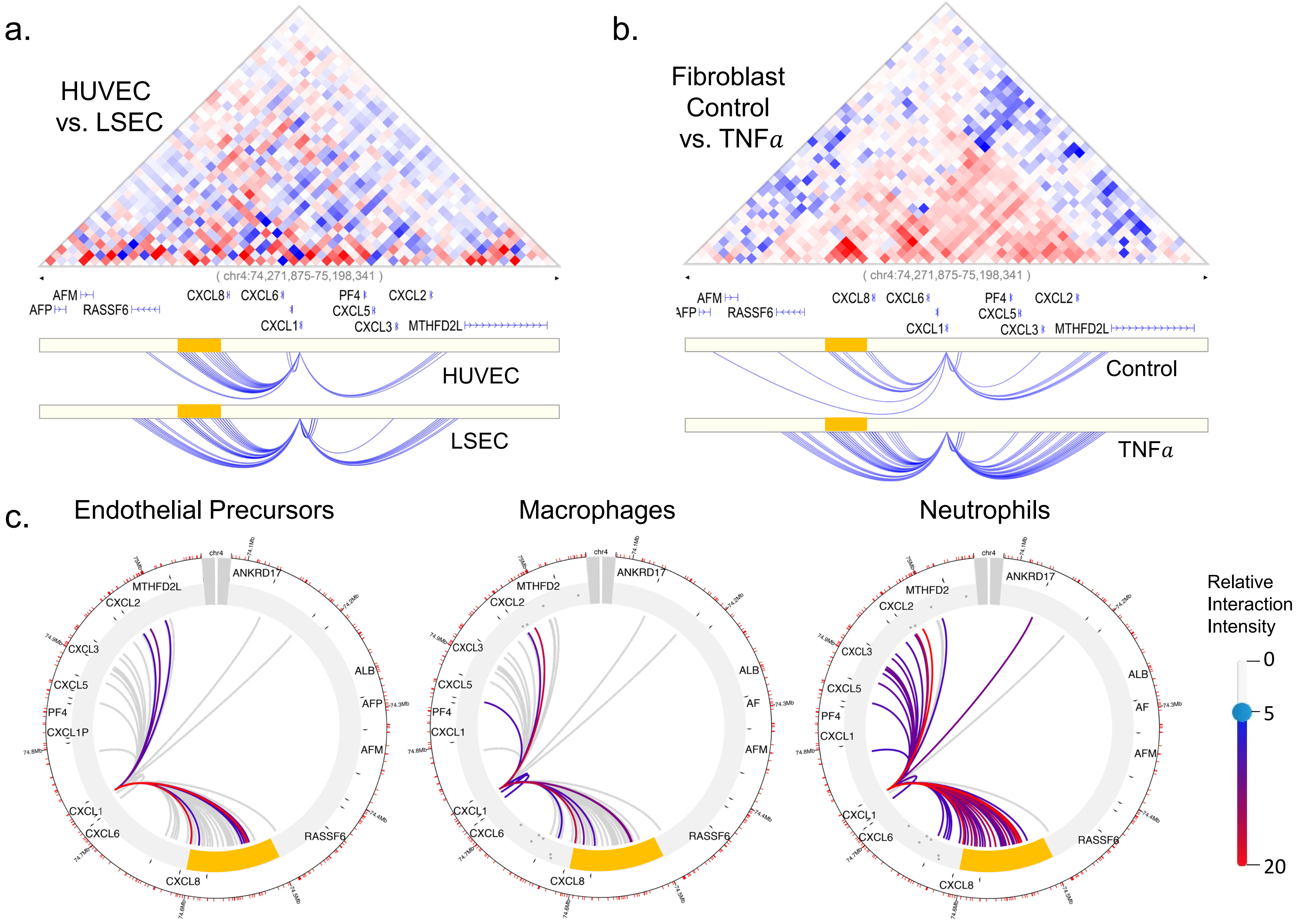

Supplementary Figure 21

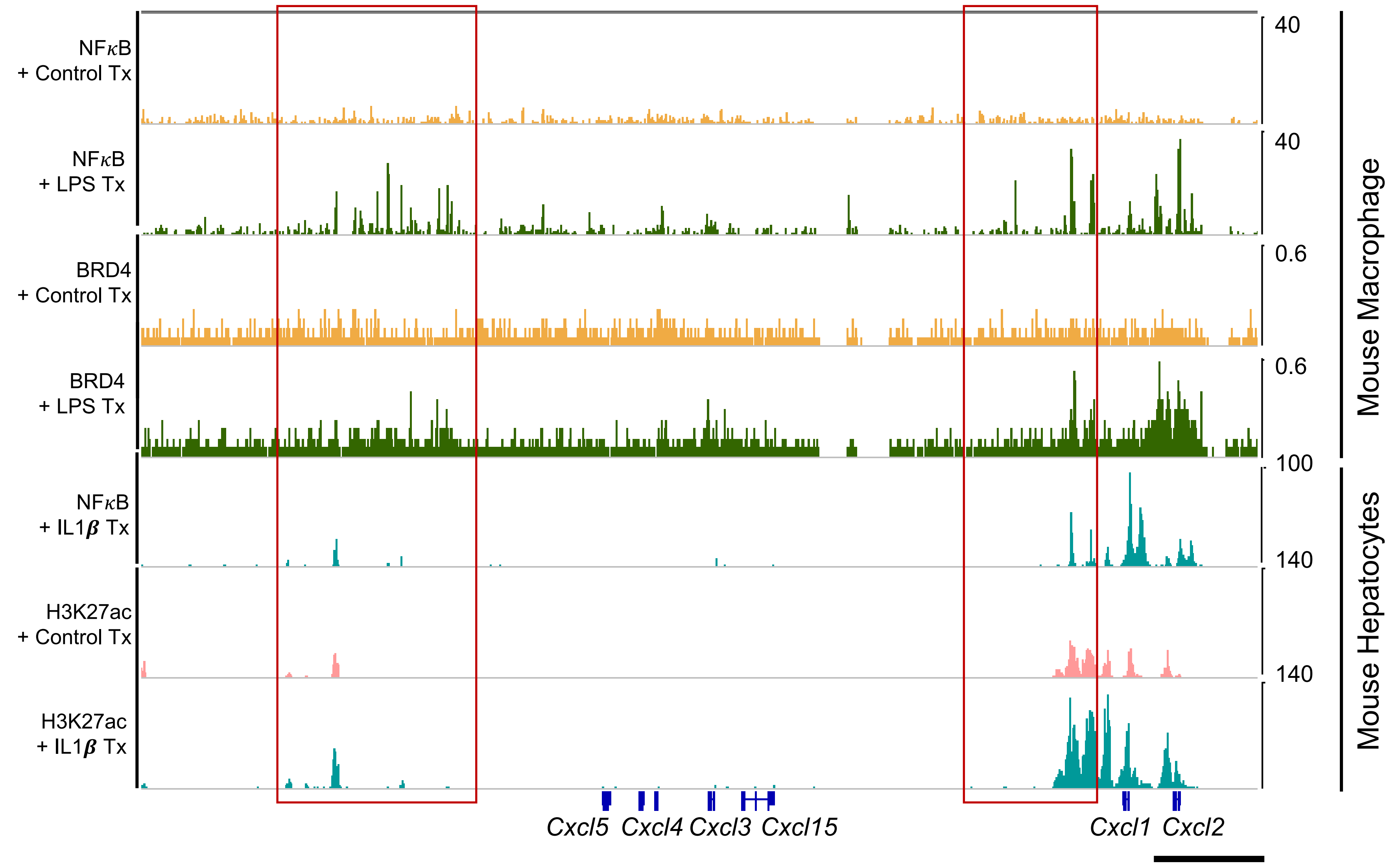

Supplementary Figure 22.

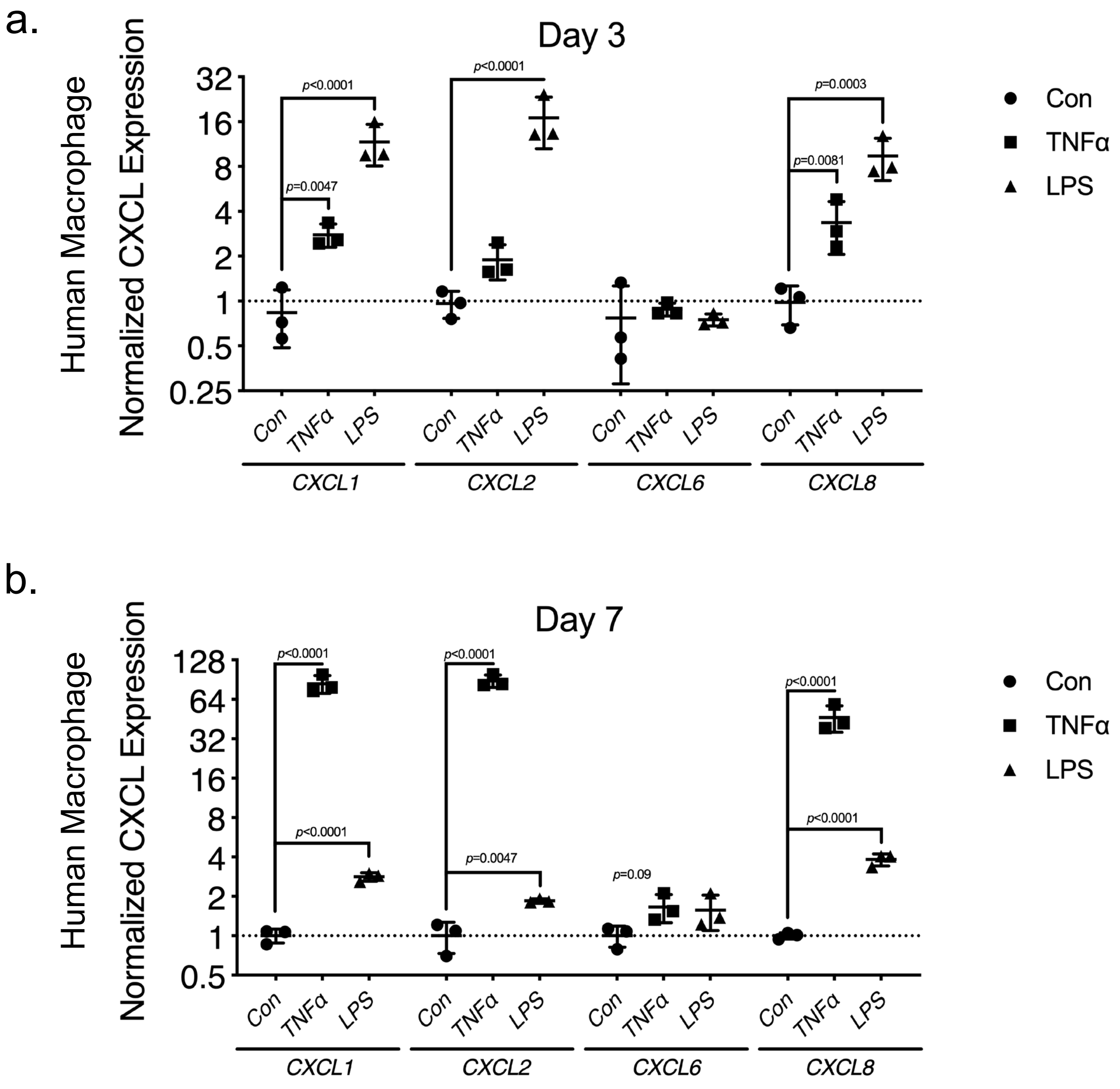

Supplementary Figure 23

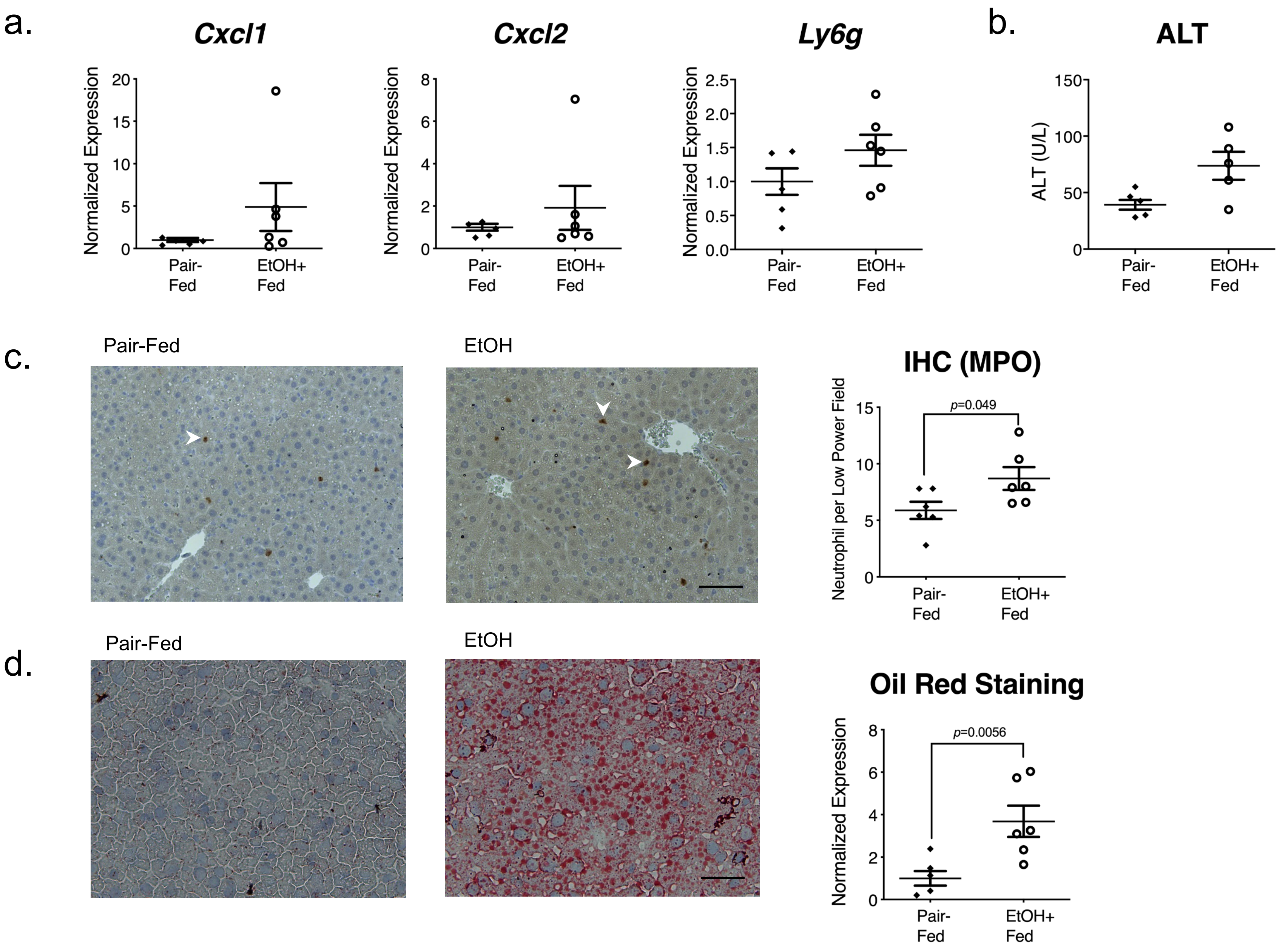

Supplementary Figure 24

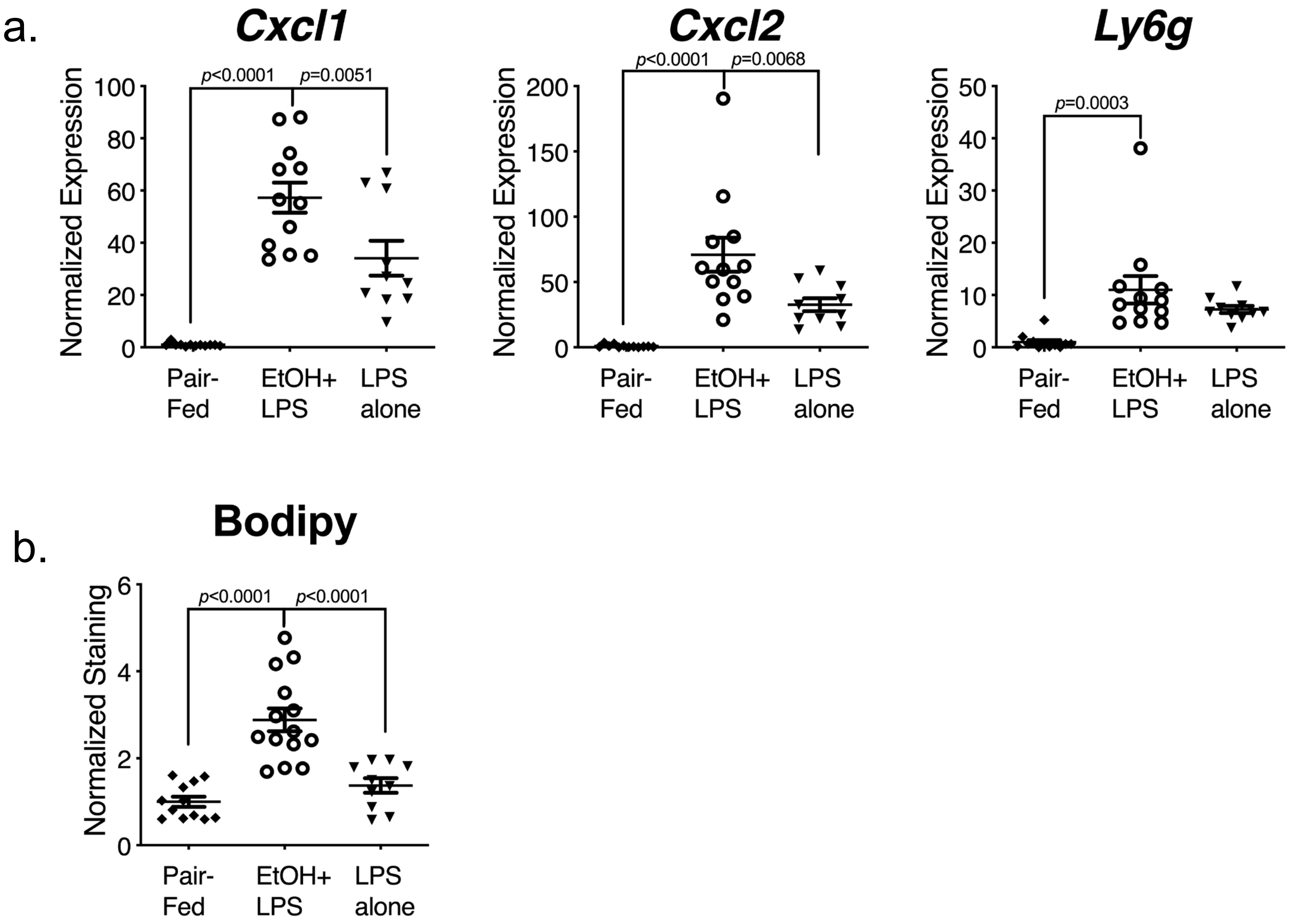

Supplementary Figure 25

a.

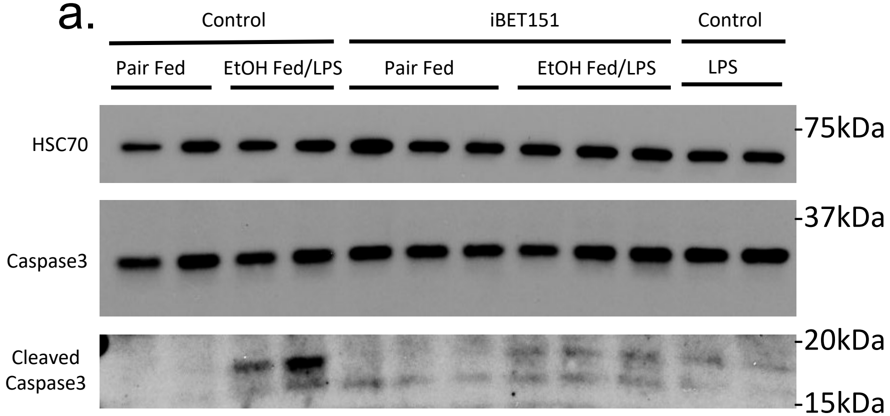

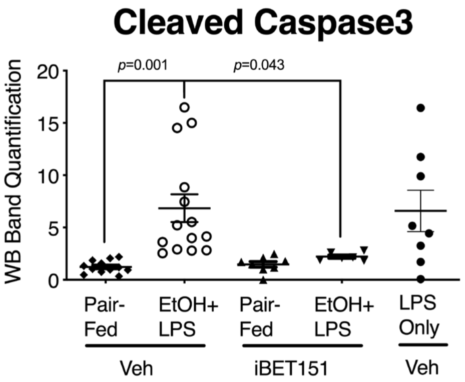

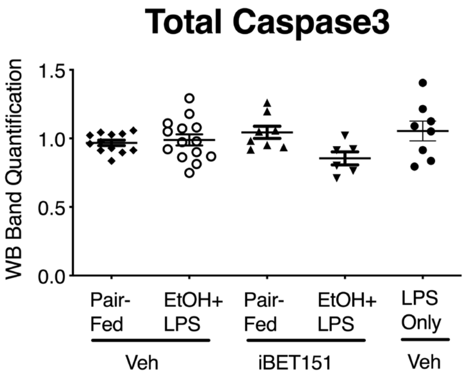

b.

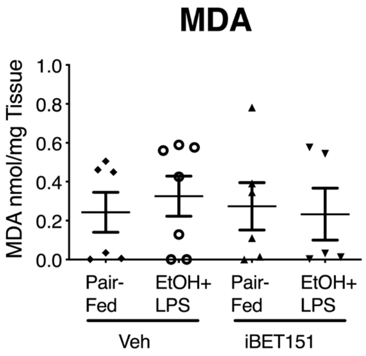

c.

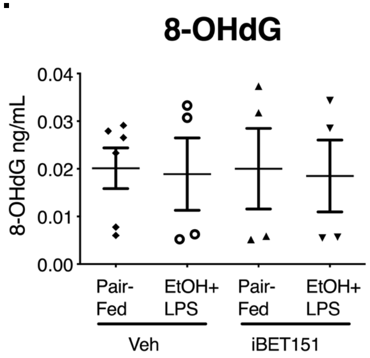

Supplementary Figure 26

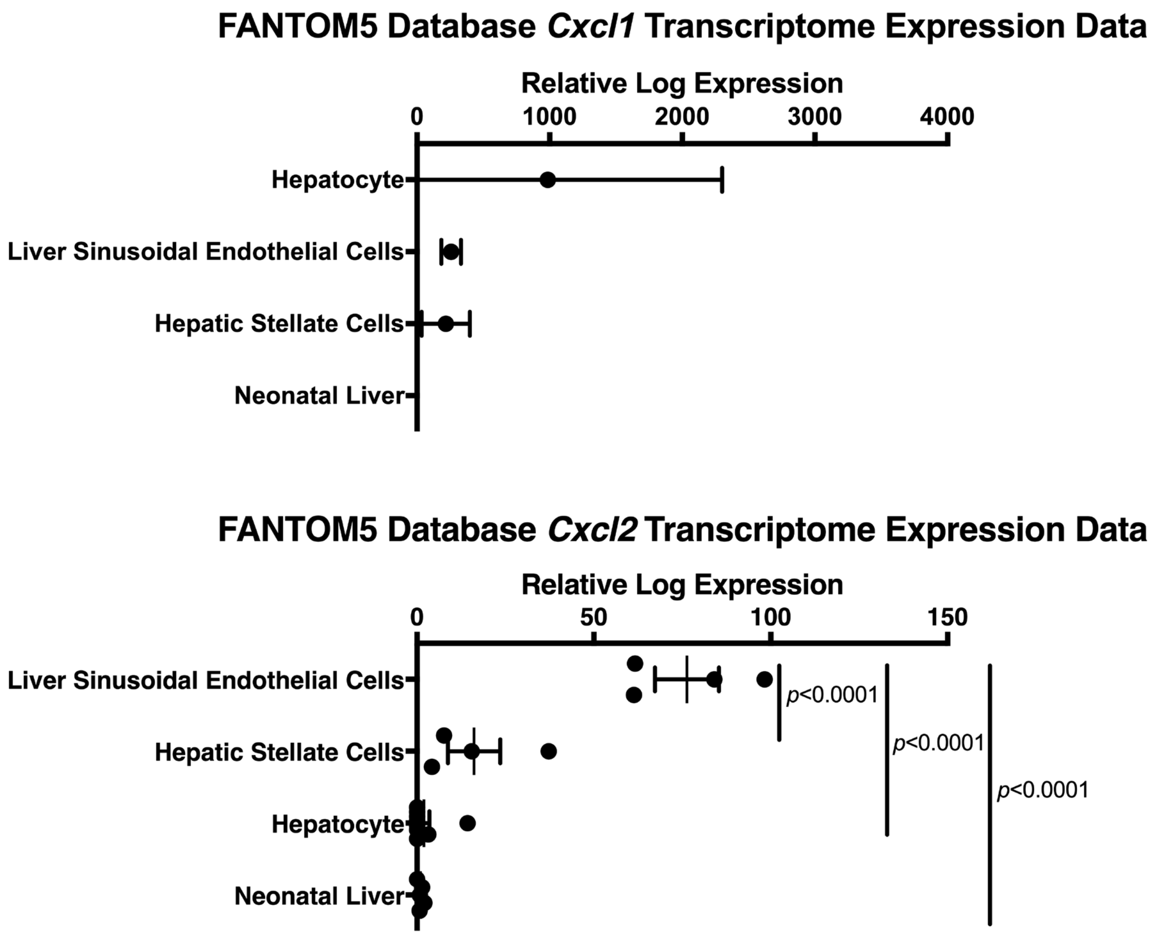

SUPPLEMENTAL FIGURE LEGENDS

Supp Figure 1| **Clinical Characteristics of AH patients.** The median and IQR of various clinical parameters are listed.

Supp Figure 2| **Principle Component Analysis of AH and Control Liver Gene Expression Profiles Demonstrate Differential Clustering.** a-c. PCA of all genes from RNA-seq showed clear separation of clinical samples into 2 clusters based on control and AH status. Expression level of differential genes with congruent histone marks from integrated analysis (b) and genes in the Granulocyte Adhesion/Diapedesis pathway (c) were analyzed and showed similar separation.

Supp Figure 3| **Heatmap of RNA-seq with Histone ChIP-seq Profiles.** a. Heatmap of RNA-seq and ChIP-seq for marks H3K4me3, H3K4me1, H3K27ac, and H3K27me3 for differentially expressed genes are plotted. ChIP-seq signal over TSS ± 2kb was estimated as counts per 10M uniquely mapped, non-redundant reads in $\log_2$ scaled and quantile normalized. Z-scores were plotted. b. The input-subtracted read density (RPM, reads per million) in 100-bp non-overlapping bins over the TSS ± 5kb region was plotted separately for the AH up- and down-regulated genes. Bin signal of all protein-coding genes was quantile normalized. There was an increase of signal from AH samples for active marks H3K27ac and H3K4me3 in AH up-regulated genes. Conversely, there was a decrease of signal from AH samples for repressive mark H3K27me3 in AH down-regulated genes.

Supp Figure 4| **Correlation of Four Histone Mark Profiles with Gene Expression Change.** The 761 genes down regulated in AH were split into three groups based on H3K4me3 status in the proximal regulatory regions: no peaks, no signal change, and reduced signals as identified by DiffBind analysis. For genes in each of the three groups, the number of genes showing the expected changes in histone modifications (i.e., increased signals for H3K27me3 and decreased signals for the other three active marks) in the proximal regulatory regions (TSS ± 2kb) and H3K27ac in the distal regulatory regions (≥ 2.5 kb away from TSS) was estimated (FDR ≤0.01 and log2 (fold change) ≥1 in DiffBind). For the 761 genes down−regulated in AH, 283 genes showed reduced H3K4me3 in the promoters compared to normal. Of the remaining 478 genes whose promoters had no H3K4me3 peaks (184 genes) or no H3K4me3 changes (294 genes), 229 (158+71) were associated with increased H3K27me3 or decreased H3K4me1/H3K27ac in the promoters, or decreased H3K27ac in the nearest enhancers. Thus, two-thirds (512/761) of the AH down-regulated genes appeared to be associated with the expected chromatin changes in the promoters or enhancers. On the other hand, for the 950 genes up−regulated in AH, 54 genes showed increased H3K4me3 in the promoters compared to normal. Of the remaining 896 genes with no H3K4me3 peaks (231) or no H3K4me3 changes (664) in promoters, 342 (281+61) were associated with decreased H3K27me3 or increased H3K4me1/H3K27ac in the promoters, or increased H3K27ac in the nearest enhancers. Together, 396 (41.7%) of the AH up-regulated genes were associated with chromatin changes in the promoters or enhancers. Distal only: the genes only associated with H3K27ac changes in the enhancers, but no changes of the 4 marks in the TSS ± 2kb regions; distal: the genes associated with H3K27ac changes in the enhancers as well as the changes of H3K27me3, H3K4me1 or H3K4me3 in the TSS ± 2kb regions.

Supp Figure 5| **Active Histone Marks are Enriched on *CXCL 1, 6,* and *8*.** The CXCL gene loci were examined in histone mark ChIP-seq. Representative AH and Control samples are shown to demonstrate that active histone marks H3K27ac and H3K4me3 were increased at these loci whereas occupancy of repressive mark H3K27me3 decreased. This correlated with increased expression seen at mRNA level (Blue: normal control liver, Red: AH liver).

Supp Figure 6| **FANTOM5 Database CXCL Chemokine Expression Levels in Normal Human Livers.** FANTOM5 human hg19 promoterome was accessed for genes *CXCL 1, 2, 6,* and *8*. Phase 1 and 2 pooled human tracks were accessed, filtered for liver related sources, and gene expression values from various tissue/cell sources were analyzed with mRNA CAGE sequencing. Values are plotted in relative log expression (RLE), and one-way ANOVA analysis was performed on the expression levels, with Post-hoc Dunnett's multiple comparison correction (n=3 for LSEC, HSC, and Hepatocyte sources and n=1 for fetal and adult liver tissues). Data represented as mean +/- SD.

Supp Figure 7| **Schematic of Predicated NF-κB Binding Sites on CXCL Promoters and Super Enhancer.** Schematic of the CXCL locus was used to demonstrate the presence of NF-κB binding motifs in the CXCL promoter regions (labeled as P with gene name) or in CXCL SE (labeled as E sequentially based on distance away from *CXCL8*). TNFα treated LSEC H3K27ac ChIP-seq track was shown to highlight positions of activated chromosomal regions. The NF-κB binding site targeted for dCas9-KRAB suppression used in subsequent analysis was labeled as E1. Scale bar represents 50 kb.

Supp Figure 8| **Microfluidic Chamber Analysis of Neutrophil Adhesion Demonstrates Increased Neutrophil in Presence of LSEC Secreted Factors.** a. Schematic of microfluidic chamber device. Cells are added to reservoir wells and flown through the chamber channels (orange canals) and drained from a connecting tubing (not shown). b. Neutrophils attached to LSECs lined chamber were quantified under various conditions. Neutrophils were labeled with Hoechst dye (round, bright blue) and LSECs could also be seen (elongated cells). Scale bar = 100μm. c. Quantification of neutrophil attachment normalized to control condition (n=4). Addition of CXCL1 (n=6) and LSEC medium (n=3) increased adhesion of neutrophils to LSECs. n represent number of chamber channels from 2 independent experiments. Experiments were performed more than 3 times with similar results. Arrows point to neutrophils. One-way ANOVA analysis was performed on the fold change ratio in cell counts, with Post-hoc Dunnett's multiple comparison correction. Data represented as mean +/- SD.

Supp Figure 9| **Transwell Neutrophil Chemotaxis Assay Demonstrates Increased Chemotaxis with TNFα Stimulated LSEC Supernatant.** a. IncuCyte obtained phase photos of LSECs (pink arrows) with attached neutrophils (white arrows) under various conditions. Scale bar = 100μm. b. Quantification of neutrophil in lower chamber (chemotactic cells) per standard image field. Addition of CXCL1 recombinant protein or LSEC cultured medium increased neutrophil chemotaxis to lower chamber. Pretreatment of LSEC with TNFα further enhances neutrophil chemotaxis, while treatment with Celastrol diminished chemotaxis in a dose-dependent manner (n=3 biologically independent samples, the experiment was replicated with similar result). One-

way ANOVA analysis was performed on neutrophil cell counts, with Post-hoc Tukey's multiple comparison correction. Data represented as mean +/- SD.

Supp Figure 10| **HEK293T Lacks Chromatin Interaction with Putative Enhancer after TNFα Stimulation.** a. 4C was performed on TNFα stimulated LSEC cells and HEK293T cells. Interactions with *CXCL1* promoter were plotted against fragment read count. A genome region of about 75 kb contained two peaks of *CXCL1* interaction under TNFα stimulation (enclosed in red box) in LSECs. No interaction was seen in HEK293T cells. Viewpoint (VP) was labeled with blackline indicating the location of the reference sequence. Scale bar represents 50 kb. b. *in silico* analysis of HUVEC ChIP-seq for H3K27ac, NF-κB and BRD4 were obtained from public database, analyzed for this locus, and plotted under control conditions (gold) or TNFα stimulation (green). Black dot indicates location of NF-κB site (E1) targeted for subsequent dCas9-KRAB experiments.

Supp Figure 11| **Identification of CXCL Super Enhancer in LSEC with H3K27ac ChIP-seq.** a. IGV snapshot of H3K27ac and NF-κB ChIP-seq signals in human LSECs and NF-κB ChIP-seq signals from HUVECs showing the CXCL locus (Gold = baseline condition, Green = TNFα treatment). H3K27ac occupancy in LSECs was enriched in the putative super-enhancer region and further increased after TNFα treatment. ChIP-seq of LSECs and HUVECs demonstrated enriched NF-κB binding in putative super-enhancer after TNFα treatment. There are lower signals of NF-κB ChIP-seq from LSECs compared to HUVECs due to technical limitations, but the most significant enrichment peaks were preserved and similar between the two groups. Black dot indicates location of NF-κB site (E1) targeted for subsequent dCas9-KRAB experiments. Scale bar represents 50 kb. b, c. ROSE algorithm of putative super-enhancer analysis from HUVEC cells without (b) or with (c) TNFα treatment. The top peaks in orange dashed box showed the most H3K27ac enrichment and are considered to be putative super-enhancers.

Supp Figure 12| **NF-κB Binding and H3K27ac Occupancy Increase with TNFα Stimulation.** a. ChIP-qPCR assays for NF-κB binding at various predicated NF-κB binding sites on CXCL promoters and SE were performed (as marked in **Supp Figure 7**). Sequence enrichment was normalized to percentage of input. There is enhancement of NF-κB binding at most of these sites with TNFα treatment (*CXCL1, 2, 3, 5, 8*, E1, and E4). n=6 for *CXCL1* and E1, n=4 for other samples. n represent biologically independent samples from 3 independent experiments. Data represented as mean +/- SD. ChIP-qPCR isotype control with IgG showed minimal binding. Two sided, paired *t* test were performed for analysis. b. ChIP-qPCR assays for H3K27ac occupancy at *CXCL1* Promoter and CXCL SE E1 site (n=3 biologically independent samples, experiment was replicated with similar result). There is enrichment of H3K27ac at both loci, which is further increased after TNFα treatment and attenuated after Celastrol treatment. Two-way matched-pairs ANOVA was performed with Post-hoc Tukey's multiple comparison correction. Data represented as mean +/- SD.

Supp Figure 13| **3C Chromatin Conformation Capture Shows Increased Interaction with TNFα Treatment That is Unchanged with CRISPR Targeting.** a. 3C experiments were performed on control (black lines) or dCas9-KRAB cells targeting *CXCL1* promoter (red lines) or CXCL SE

(yellow lines) under control (solid lines) and TNFα treatment (dash lines) to detect binding of predicted CXCL super-enhancer with promoters of various CXCLs. E1 site (black dot) within the CXCL super-enhancer (vertical dash lines) was used as reference sequence. Interaction frequencies were plotted after normalized to that of *RASSF6*, a nearby noninflammatory gene used as control. Multiple other gene sequences between target CXCL promoters were selected as additional controls. X-axis maps relevant gene sequences as kb distance from *RASSF6*. Three-way ANOVA analysis was performed on the relative interaction frequencies separately with Control vs. dCas9-KRAB sgCXCL1 Promoter and Control vs. dCas9-KRAB sgCXCL SE, followed by Post-hoc Dunnett's multiple comparison correction (n=3 from 3 independent experiment). There was no significant change to SE-promoter interactions with either TNFα treatment or dCas9-KRAB targeting. There are increased gene interactions at *CXCL8* and *CXCL1* compared to negative control. Data represented as mean +/- SD.

Supp Figure 14| **Selection of sgRNA for CRISPR dCas9-KRAB Targeting.** dCas9-KRAB fusion protein targeting CXCL super-enhancer NFκB site suppressed CXCL expression in LSECs. 15 sgRNAs targeting one of four top predicted NFκB binding sites on the CXCL super-enhancer were studied here in dCas9-KRAB treated cells. For simplicity of presentation, the sgRNAs with best activity targeting each NFκB binding site was shown alongside with control sgRNA (green box) and *CXCL1* promoter sgRNA (red box). SE sgRNA decreased *CXCL1, 3, 6,* and *8* expression to varying degrees (qPCR), but did not affect expression of *MTHFD2L*, a nearby noninflammatory gene (negative control). Of these sgRNAs targeting CXCL SE, sgRNA targeting E1 showed best efficacy and consistency of CXCL deduction and was used for subsequent analysis (yellow box). n= 4 biologically independent samples from 2 experiments. Changes in chemokine expression were calculated as fold change over basal expression and $\log_{10}$ (fold change) was plotted on the y-axis. Two-way ANOVA analysis was performed on the log-transformed ratios followed by Post-hoc Sidak's multiple comparison correction. Data represented as mean +/- SD.

Supp Figure 15| **CRISPR dCas9-KRAB LSEC Cytotoxicity Assay.** IncuCyte system was utilized to image cells and assess for cell toxicity. Dying cells (white arrows) are stained green by fluorescent dye, and green cells are counted for each low power image field. Images shown were acquired after 4hrs of incubation with dye. Quantification for the number of dead cells was done at 6hr intervals for 24hr, showing no difference among control, P1 sgRNA/dCas9-KRAB cotransfected cells, and E1 sgRNA/dCas9-KRAB cotransfected cells. Data represented as mean +/- SD, n=3 biologically independent samples, experiment was repeated with similar results. Two-way RM ANOVA analysis showed no significant difference among treatment groups. Scale bar = 100μm.

Supp Figure 16| **dCas9-KRAB Fusion Protein Targeting CXCL Super Enhancer NFκB Site Suppressed CXCL Expression in LSECs.** a. dCas9-KRAB treatment with sgRNA targeting CXCL super-enhancer (E1) decreased *CXCL1, 6,* and *8* expression, but did not affect expression of *CXCL2, 3, 5*, and *MTHFD2L*, a nearby noninflammatory gene (negative control). Log_{10} (fold change) was plotted, n=4 biologically independent samples from 2 experiments. Data represented as mean +/- SD. b. dCas9-KRAB treatment with sgRNA targeting *CXCL1* promoter (P1) decreased expression of CXCL1, but not other CXCLs by qPCR, n=4 biologically independent

samples from 2 experiments. Data represented as mean +/- SD. c, d. sgRNA treatment alone targeting either *CXCL1* promoter or CXCL SE without dCas9-KRAB did not result in significant repression of CXCL expression. Two-way matched-pairs ANOVA was performed on log-transformed fold-change values with Post-hoc Tukey's multiple comparison correction. Data represented as mean +/- SD, n=4 biologically independent samples from 2 experiments. All data were repeated at least 3 times with similar results.

Supp Figure 17| **dCas9-FLAG Transduced Cells Suppressed CXCL Expression.** a. Cotransfection of dCas9-FLAG with E1 sgRNA decreased expression of *CXCL1* (n=7), and but not *CXCL 6* (n=4), *CXCL8* (n=6), or *MTHFD2L* (n=6) by qPCR, n represents biologically independent samples from 3 experiments. Changes in chemokine expression were calculated as fold change over basal expression, and $\log_{10}$ (fold change) was plotted on the y-axis. Data represented as mean +/- SD. B. Cotransfection of dCas9-FLAG with P1 sgRNA decreased *CXCL1* (n=7) expression at basal level and after TNFα treatment. There was no effect on expression of *CXCL6* (n=4), *CXCL8* (n=6), or *MTHFDL2* (n=4), n represents biologically independent samples from 3 experiments. Two-way matched-pairs ANOVA was performed on log-transformed fold-change values, with Post-hoc Tukey's multiple comparison correction. Data represented as mean +/- SD.

Supp Figure 18| **Bromodomain Inhibitors Suppress Expression of CXCLs.** a and b, LSECs were pretreated with iBET151 (0-50μM) (a, n=4) or UMN627 (0-50μM) (b, n=4). *CXCL2, 3, and* 5 expression levels were assessed by qPCR. Expression levels were normalized to basal condition and $\log_{10}$ fold change values were plotted. n represents biologically independent samples from 2 experiments. One-way matched-pairs ANOVA analysis was performed with Post-hoc Dunnett's multiple comparison correction. Data represented as mean +/- SD. There were linear trends for decreasing *CXCL2, 3, 5* expression with increasing iBET151 and UMN627 concentrations with TNFα, and with increasing iBET151 concentrations without TNFα ($p<0.01$ for all groups).

Supp Figure 19| **iBET151 LSEC Cytotoxicity Assay.** IncuCyte system was utilized to image cells and assess for cytotoxicity. Dying cells (white arrows) are stained green by fluorescent dye, and green cells are counted for each low power image field. Cells were pretreated with control medium or iBET151 and/or TNFα, and quantification for the percentage of dead cells was done at 30mins intervals for 10hr starting at 15mins. Images shown were acquired after 4hrs of incubation. Data represented as mean +/- SD, n=6 for Control and TNFα, and n=3 for iBET151 treatment groups. n represents biologically independent samples. Two-way RM ANOVA analysis was performed showing no significant difference among treatment groups. Scale bar = 100μm.

Supp Figure 20| **Multiple Human Cell Types Demonstrate Looping Interactions Between CXCL Super Enhancer and CXCL Promoters.** a. Comparison of HUVEC and LSEC in the levels of *cis*-interactions involving *CXCL1* promoter. Heatmap represented the differences in chromosomal interaction frequency (red, higher signal in LSECs; blue, higher signal in HUVEC), showing a similar interaction profile between the two cell types. Arc graphs represented chromosomal interactions of *CXCL1* promoter, most notably with CXCL super-enhancer (gold bar) in the two cell types. Data were accessed from the 3DIV Hi-C database. b. Comparison of *CXCL1* promoter chromosomal interactions under control condition and TNFα stimulation in IMR90 fibroblast as

determined by Hi-C. Heatmap represented the differences in chromosomal interaction with or without TNFα treatment (red, higher signal in TNFα treated cells), which showed increased cis-interactions following TNFα treatment. Arc graphs depicted chromosomal interactions of the *CXCL1* promoter, which were enhanced after TNFα stimulation, particularly with the CXCL super-enhancer (gold bar). Data were accessed from 3DIV. c. Comparison of chromosomal interactions involving *CXCL1* promoter across three primary human cell types. Elevated chromosomal interactions were identified with the CXCL super-enhancer. Capture Hi-C data were from the CHiCP web browser (https://www.chicp.org). In the three plots, CXCL super-enhancer was marked in gold color. All GSE accession numbers of data files can be found in **Supp. Table 5**.

Supp Figure 21| **The CXCL Locus Contained Putative Super Enhancers in Mouse Macrophage and Hepatocyte.** IGV snapshot of NF-κB, BRD4, and H3K27ac ChIP-seq signals in mouse macrophage and hepatocyte. The two putative super-enhancers (enclosed by red box) were identified. LPS treatment of macrophage cells increased the binding of NF-κB and BRD4 at this locus (Gold = baseline condition, Green = LPS treatment). ChIP-seq of mouse hepatocytes demonstrated enriched NF-κB binding in putative super-enhancer after IL1β treatment. H3K27ac occupancy in hepatocytes was enriched in the putative super-enhancer region and further increased after IL1β treatment (pink, baseline condition; teal, IL1β treatment). Scale bar represents 20 kb. All GSE accession numbers of datafiles can be found in **Supp. Table 5**.

Supp Figure 22| **TNFα and LPS Treatments of Human Peripheral Blood Monocytes Derived Macrophages Significantly Increased Expression of CXCL Chemokines by qPCR.** Monocytes were cultured with M-CSF to induce differentiation into macrophages. Cells at Day 3 (a) and 7 (b) of culture were assayed separately. CXCL chemokines expression are shown as fold change over control condition for qPCR in log scale. n=3, where n represents biologically independent samples. One-way ANOVA analysis of log transformed ratios was performed with Post-hoc Dunnett's multiple comparison correction. Data represented as mean +/- SD.

Supp Figure 23| **Alcohol Feeding Increased Neutrophil Infiltration in NIAAA Chronic Binge Alcohol Feeding Model.** a. qPCRs of CXCL chemokine expression and neutrophil marker *Ly6g* with alcohol feeding. Expression levels were normalized to average expression of pair-fed control mice. Data represented as mean +/- SD. b. Serum ALT levels were shown. Data represented as mean +/- SD. No significance was seen between groups for a or b. c. IHC for MPO (neutrophil marker) showed a significantly increased amount of neutrophil infiltration with alcohol feeding. Data represented as mean +/- SD. Scale bar = 100μm. d. Frozen sections of mouse liver were stained with Oil-Red-O (red). Hematoxylin counterstain was used to stain nuclei (blue). Quantification showed an increase in steatosis with alcohol. Data represented as mean +/- SD. Scale bar = 100μm. For all analysis one-way ANOVA was performed on log-transformed normalized expression values or cell counts for IHC staining or ALT values, with Post hoc Tukey's multiple comparison correction used for above analysis. N=6 for each group, except for alcohol-fed mice ALT measurement n=5 (serum was unable to be collected for 1 mouse for technical reasons).

Supp Figure 24| **Comparison of LPS Alone Treatment with Combination Alcohol-feeding/LPS Treatment Mice.** a. qPCRs of CXCL chemokine and neutrophil marker *Ly6g* were normalized to

average expression of Pair-fed control mice (n=12). Increased *Cxcl1* and *Cxcl2* expression was seen in combination group (n=12) compared to LPS alone (n=10). No significant difference was seen with *Ly6g*. Data represented as mean +/- SD. b. BODIPY 493/503 staining of mice liver was performed. Scatter plot shows normalized quantification of BODIPY 493/503 staining to Pair-fed mice (n=12 for Pair-fed, n=14 for EtOH+LPS, and n=10 for LPS only). One-way ANOVA was performed with qPCR fold changes or BODIPY quantification ratios, with Post-hoc Sidak's multiple comparison correction. Data represented as mean +/- SD.

Supp Figure 25| **Alcohol feeding/LPS Injection Increased Cleaved Caspase 3 in Mice Livers.** a. Representative Western Blotting with anti-caspase3 antibody showed increased cleaved caspase 3 in livers of alcohol-fed/LPS mice. This increase was attenuated by iBET151 injection. There is no difference to total Caspase 3 levels (n=12 Pair-fed/Veh, n=14 EtOH+LPS/Veh, n=8 Pair-fed/iBET151, n=6 EtOH+LPS/iBET151, n=8 LPS only). Quantification of the bands were shown. Data represented as mean +/- SD. b, c. Quantification of Malondialdehyde (MDA) assessing lipid peroxidation (b) (n=6 Pair-fed/Veh, n=7 EtOH+LPS/Veh, n=6 Pair-fed/iBET151, n=5 EtOH+LPS/iBET151), and 8-Hydroxy-2'-deoxyguanosine (8-OHdG) assessing DNA oxidation (c) (n=6 Pair-fed/Veh, n=4 EtOH+LPS/Veh, n=4 Pair-fed/iBET151, n=4 EtOH+LPS/iBET151) in mice livers showed no difference among treatment groups. One-way ANOVA analysis was performed with Post-hoc Dunnett's multiple comparison correction. Data represented as mean +/- SD.

Supp Figure 26| **FANTOM5 Database CXCL Chemokine Expression Levels in Normal Mouse Livers.** FANTOM5 mouse mm9 promoterome was access for genes *Cxcl1* and *2*. Phase 1 and 2 pooled mouse tracks were accessed, filtered for liver related sources, and gene expression values from various tissue/cell sources were analyzed. Values are plotted in relative log expression (RLE), and one-way ANOVA analysis was performed on the expression levels, with Post-hoc Dunnett's multiple comparison correction (n=4 for LSEC and HSC, n=9 for Hepatocyte). Data represented as mean +/- SD.

SUPPLEMENTAL METHODS

RNA-seq and Histone Mark ChIP-seq Data Analysis

RNA-seq data were analyzed using the MAP-RSeq pipeline [1]. In brief, paired-end reads were aligned to the human genome reference hg19 using TopHat (v2.1.0) [2] and gene counts were estimated using the featureCounts (v1.4.6) software [3] based on the Ensembl gene definition files. Gene expression was quantified as reads per kilobase per million mapped reads (RPKM). Protein-coding genes with RPKM >=1 in at least one sample were extracted and the top 5,000 genes with the largest between-sample variation were used in hierarchical clustering. A subset of protein-coding genes with reads per million (cpm) >=1 in at least two samples were selected for differential analysis. The differentially expressed genes between the 6 AH and 4 normal were identified using the edgeR package (v3.18.1) with the option of the trimmed mean of M-values (TMM) normalization, at the cutoffs of FDR <=1% and an absolute log2 (fold change) >=1.5.

ChIP was done in the Epigenomics Development Lab using antibodies against histone marks H3K27ac, H3K4me1, H3K4me3, and H3K27me3 as previously described with human liver tissue and against H3K27ac and NF-κB [4]. Briefly, tissue (50 mg) is homogenized for 15 – 30 seconds in 500 μl of 1X PBS using tissue grinder. Homogenized tissues or tissue culture cells were cross-linked to final 1% formaldehyde for 10 min, followed by quenching with 125 mM glycine for 5 min at room temperature and by washing with TBS. The pellets were resuspended in cell lysis buffer (10 mM Tris-HCl, pH7.5, 10 mM NaCl, 0.5% NP-40) and incubated on ice for 10 min. The lysates were aliquoted into 2 tubes and washed with MNase digestion buffer (20 mM Tris-HCl, pH7.5, 15 mM NaCl, 60 mM KCl, 1 mM CaCl2) once. After resuspending in 250 μl of the MNase digestion buffer with proteinase inhibitor cocktails for each tube, the lysates were incubated in the presence of 1,000 Gel units of MNase (NEB, M0247S) per 4 x 106 cells at 37 °C for 20 minutes with continuous mixing in thermal mixer (Fisher Scientific, 05-450-206). After adding the same volume of sonication buffer (100 mM Tris-HCl, pH8.1, 20 mM EDTA, 200 mM NaCl, 2% Triton X-100, 0.2% Sodium deoxycholate), the lysates were sonicated for 15 min (30 sec-on / 30 sec-off) in Diagenode bioruptor and centrifuged at 15,000 rpm for 10 min. The cleared supernatant equivalent to the cellularity of 4 x 10^6 cells was incubated with 2 μg of modification-specific antibodies on rocker overnight. The following antibodies are used in the experiment; anti-H3K27ac antibody (Cell signaling, 8173), in-house generated anti-H3K4me3 antibody (EDL lot 1), in-house generated anti-H3K4me1 antibody (EDL lot 1), anti-H3K27me3 antibody (Cell signaling, 9733), and anti-NF-κB (RELA subunit) (Cell signaling 8242S). For the next-generation sequencing, ChIP-seq libraries were prepared from 10 ng of ChIP and input DNAs with the Ovation Ultralow DR Multiplex system (NuGEN). The ChIP-seq libraries were sequenced to 51 base pairs from both ends using the Illumina HiSeq 2000 in the Mayo Clinic Medical Genomics Core.

ChIP-seq data was analyzed using the HiChIP pipeline [5]. Briefly, paired-end reads were mapped to the hg19 genome reference using Burrows-Wheeler Alignment (BWA) [6]. Pairs of reads with one or both ends being uniquely mapped were retained and duplicates were removed using Picard MarkDuplicates command (http://broadinstitute.github.io/picard/). Narrow peaks from H3K4me3, H3K4me1 and H3K27ac were identified using model-based analysis of ChIP-seq (MACS2) software package at FDR <=1% [7]. Broad peaks from H3K27me3 were identified using spatial

clustering approach for the identification of ChIP-enriched regions (SICER) software package at FDR <=1% [8]. For data visualization, Bedtools [9] together with in-house scripts were used to generate per-million read density profile at 200-bp window size and a step size of 20 bp. ChIP-seq signal tracks were visualized in Integrative Genomics Viewer (IGV) software.

For each of the ChIP-seq libraries, the number of reads in the TSS ± 2kb region of all protein-coding genes was estimated, normalized to 10 million uniquely mapped reads (RP10M), log2 transformed and quantile normalized across samples. The normalized values extracted for the differentially expression genes were used to generate the heatmaps. In addition, the read density (RPM, reads per million) in 100-bp non-overlapping bins over the TSS ± 5kb region was calculated using the ngs.plot tool (v2.02) [10]. The input-subtracted read density was plot separately for the AH up- and down-regulated genes. Finally, for each of the histone marks, peaks present in at least two samples were retained and those showing increased or decreased signals in AH relative to normal were identified by the DiffBind package (v2.4.8) at the FDR <=1% and absolute fold change >=2. The retained peaks were assigned to the proximal (TSS ± 2kb) or to the nearest distal regulatory regions outside of TSS ± 2.5kb. Upregulated genes with increased signals of active marks (H3K4me3, H3K4me1 and H3K27ac) or decreased signal of H3K27me3, and downregulated genes with the reverse histone mark patterns were deemed to be genes of interest in the integrated analysis.

A heatmap was generated from the Z-scores of expression FPKM values with genes in the integrated analysis. Z-scores were calculated by subtracting the mean FPKM values in all subjects and dividing by the standard deviation. Genes in the integrated analysis were analyzed with Ingenuity pathway analysis (IPA) to uncover common regulatory pathways. Upregulated genes from the integrated analysis were analyzed separately. A canonical pathway unrelated to the liver (sperm motility) was removed, and top differentially activated pathways along with predicated upstream regulators were displayed. To explore the potential roles of TNFα and NF-κB signaling in AH differentially expressed genes, gene set enrichment analysis (GSEA) were performed using default settings (1000 permutations and for a maximum size of sets of 1000) on AH differentially upregulated genes as previously described [11]. TNFα/NF-κB pathway target genes were obtained from previously published gene sets [12,13]. To examine the within-group variations of AH and normal subjects enlisted in the study, principle component analyses (PCA) were performed separately for the whole transcriptome, differential genes in the integrated analysis, and genes in the granulocyte and agranulocyte adhesion and diapedesis pathways. Expression level (RPKM values) of all cataloged genes were filtered and lowly expressed genes (<1 RPKM) were removed. Z scores were calculated from RPKM, and data was analyzed with built-in R function prcomp and plotted with package ggplot2 and rgl.

Super enhancer calling was performed using H3K27ac data of LSEC and HUVEC cells [14] using the ROSE tool (v1.0.0, http://younglab.wi.mit.edu/super_enhancer_code.html) [15,16], using H3K27ac peaks called with the Model-based analysis of ChIP-Seq (MACS) software package (v2.0.10) [7]. Peaks within TSS +/- 2.5kb were excluded to account for promoter biases, and the remaining peaks within a distance of 12.5kb or less were stitched together.

Human Liver Cell RNA-seq

Human primary liver cells used for RNA-seq were purchased from ScienCell. Human LSECs were isolated from mixed primary cultures containing all liver cells by CD31 antibody and characterized by immunofluorescence with antibodies specific to vWF/Factor VIII and CD31 (PECAM). LSECs are negative for HIV-1, HBV, HCV, mycoplasma, bacteria, yeast and fungi. RNA-seq was performed and analyzed in the same manner as described above for human liver RNA-seq. The RPKM values of CXCL chemokines were extracted and normalized to multiple house-keeping genes (C1orf43, CHMP2A, GPI, PSMB2, PSMB4, RAB7A, VCP, and VPS29) as previously described [17]. FANTOM5 database RNA-seq results are cited in this study, and FANTOM5 technical support team has also indicated that cells used in their analysis were also purchased from ScienCell.

Analysis of Public Hi-C Data of Human Cells

Hi-C data in HUVEC (GSM1551629), LSEC (ENCLB284TIY) and fibroblasts (GSM1055800 and GSM1055802) were accessed through the 3DIV Hi-C database [18]. Heatmap shows the differences in normalized interaction frequency between the indicated cell types at the *CXCL1* locus (**Supp. Figure 12A, 12B**) [19-21]. Promoter Capture Hi-C (CHi-C) data of human endothelial precursors, macrophages, and neutrophils were accessed from the CHiCP web browser [22], using data published by Javiette, *et. al* [23]. Datasets used in the study listed in **Supp Table 5.**

Analysis of Public scRNA-seq Data

scRNA-seq data of mouse and human liver were analyzed in **Supp Table 1**. Data was downloaded from GEO database and analyzed in Seurat V4 [24]. In studies by MacParland et.al. (GSE115469) [25] and Aizarani et.al. (GSE124395) [26], clustering information of single cells were provided by the authors. In studies by Ramachandran et.al. (GSE136103) [27], Xiong et.al. (GSE129516) [28], and Nault et.al. (GSE148339) [29], clustering information was not available. Data matrixes were analyzed in Seurat, and cell clustering was performed with marker genes provided by each respective study. For each study, the total count of CXCL chemokines transcript was tallied per cell type and relative contribution of each cell type was calculated as a percentage and reported in **Supp Table 1**.

Quantitative Real-Time Polymerase Chain Reaction (PCR)

RNA extraction was performed with RNeasy kit (Qiagen 74104) from cells and mouse tissue according to the manufacturer's instructions. RNA Quantification was performed with spectrophotometry (NanoDrop, Thermo Scientific). 500 ng of mRNA was used for cDNA synthesis with dNTP and oligo primer using SuperScriptTM III (Invitrogen 18080-093) for reverse transcription per the manufacturer's protocol. Real-Time PCR was performed from cDNA using IQ SYBR Green Mix (Biorad 1725121) on the 7500 Real-Time PCR system (Applied Biosystems), according to the manufacturer's instructions. Amplification of GAPDH and β-actin was performed for respective samples as internal controls. Each experiment was done in duplicates. Primer sequences are listed in **Supp Table 1**.

Neutrophil Chemotaxis Assays

Neutrophil Isolation: Human neutrophils were isolated from whole blood using an immunomagnetic separation technique (Miltenyl Biotec 130-104-434 and MACSxpress Separator)

according to manufacture protocol. Following isolation, cells were suspended in RPMI (Gibco 1640).

Microfluidic Device fabrication: Microfluidic devices were fabricated using standard soft lithography approaches. Design of the device with 6 parallel channels was created in AutoCAD and converted into photomask by CAD/Art services (Bandon, Oregon) (**Supp. Figure 8A**). Microfluidic channels were then molded in polydimethyl siloxane (PDMS) and secured atop a 3 × 1 in. glass slide. A cloning cylinder was mounted at the inlet of each of the six channels and was used for loading neutrophils. Prior to seeding cells, microfluidic chambers were infused with 10% FBS for 30 minutes. Subsequently, FBS was removed and devices were washed twice with ice cold PBS and coated with collagen. LSECs were seeded into devices and cultured for 2 days prior to use.

Chamber Chemotaxis Experiment: Neutrophils were stained with Hoechst dye (Invitrogen 33342) for visualization and then infused into a microfluidic device containing LSECs for 10 min at the flow rate of 3.84 μL/min (shear stress of 0.02Pa) for 10 min. In select samples, neutrophils were resuspended in medium supplemented with recombinant human CXCL1 (STEMCELL 78063.1) at 100 ng/mL or in conditional LSEC medium (medium exposed to LSECs for 24 hours). Afterwards, microfluidic devices were washed by flowing PBS for additional 10 min. The neutrophils attached in microfluidic channels were quantified by acquiring images at 10x magnification, 5 images per channel.

Transwell Chemotaxis Experiment: LSECs were seeded in Transwell plates (Corning 3421) and cultured for 24hrs with 600μL of basal, TNFα (10ng/mL) supplemented medium, or TNFα and Celastrol supplemented medium. Prior to the experiment, overnight medium was removed from the negative (basal medium) and positive (recombinant CXCL1) controls and appropriate fresh medium was added. 600 μL of endothelial medium was added back to each control well, and recombinant CXCL1 at 100ng/mL was added to positive control wells. Isolated neutrophils (1 million cells) were added to each well insert. Chemotaxis experiment was allowed to occur at 37°C for 1 hr. Inserts were removed and plates were imaged in IncuCyte at 10x magnification with 5 visual fields. Quantification of neutrophils were done manually.

4C-Seq Methods and Data Analysis

10 million LSEC cells with or without TNFα treatment or HEK293T (ATCC CRL-11268) cells were fixed in 1X PBS/10%FCS, 2% (w/v) final concentration formaldehyde for 10 min at room temperature. A final 0.125M Glycine was added to stop the fixation on ice, followed by 400g x8min in 40C to remove supernatant. Pellets were resuspended in cold lysis buffer (50 mM Tris-HCl pH 7.5, 150 mM NaCl, 5 mM EDTA, 0.5% NP-40, 1% TX-100 and 1× complete protease inhibitors (Roche #11245200)) and incubate 10 min on ice. The efficiency of cell lysis was determined by Methyl Green-Pyronin staining (Sigma #HT70116). The DNA was digested with Csp6I (New England BioLabs R0639) and NlaIII (New England BioLabs R0125) as primary and secondary enzymes, respectively. T4 DNA ligase (New England BioLabs M0202) was used for both ligation steps. Specific primers were designed at the CXCL1 gene promoter with 4C-CXCL1 reverse primer (Csp6I) and 4C-CXCL1 forward primer (NlaIII). PCR amplifications were made with Expand Long Template PCR System (Roche). The bar-coded DNA libraries were generated with Illumina

primers for each sample and purified with a High Pure PCR Product Purification Kit (Roche) and sent for deep sequencing. The libraries were sequenced on an Ilumina HiSeq 2000 instrument (Illumina, CA, USA) as per Mayo Clinic Center for Individual Medicine Medical Genomics Facility. 4C libraries were sequenced to 100 bp from both ends. Primer sequences were trimmed off using the Trim Galore package (v0.2.2, https://www.bioinformatics.babraham.ac.uk/projects/trim_galore/). We only retained the pairs of reads whose primer sequences were trimmed. The retained reads were mapped to the human genome reference hg19 using BWA-MEM (v0.7.10) 9 (https://arxiv.org/abs/1303.3997) in single-end mode. The mapped reads were filtered to keep those with a minimal mapping quality score of 20. Cis interactions within 1 Mb of the reference region were identified using the R package Basic4Cseq[30]. Illumina adaptors were included in the primer sequences.

Additional 3C-qPCR Methods and Data Analysis

10 million LSEC cells or CRISPR dCas9-KRAB cells (generated with methods provided below) with or without TNFα treatment was trypsinized and resuspended in 10% FBS/DMEM in single-cell suspensions. Cells were crosslinked with in 1X PBS/10%FCS, 2% (w/v) final concentration formaldehyde for 10 min at room temperature followed by addition of 0.125M Glycine to stop the fixation on ice. Cells were pelleted and resuspended in cold lysis buffer (50 mM Tris-HCl pH 7.5, 150 mM NaCl, 5 mM EDTA, 0.5% NP-40, 1% TX-100 and 1× complete protease inhibitors (Roche #11245200)) and incubated for 10 min on ice. The efficiency of cell lysis was determined by Methyl Green-Pyronin staining (Sigma #HT70116). DNA was digested with NlaIII restriction enzyme (New England BioLabs R0125) overnight, and ligated with T4 DNA ligase (New England BioLabs M0202). DNA was then de-crosslinked overnight and purified with phenol-chloroform. A NlaIII restriction site near the predicted NF-κB binding site in the CXCL super-enhancer was used to design the reference sequence. Specific primers were designed to detect DNA fragment ligation at various restriction sites near each CXCL promoter NF-κB binding site and control segments *RASSF6* and intronic segments between CXCL genes. The sequences of primers used to detect ligated segments are provided in **Supp Table 4**. The interaction frequencies of various segments were assessed by probe-based qPCR (PrimeTime, IDT), and amplification levels were normalized to *RASSF6* and plotted (**Figure 3f, Supp Figure 13**).

Additional Cell Culture Techniques

LSECs were thawed and cultured according to standard cell culture conditions and according to manufacture instruction (ScienCell). Briefly, LSECs are plated at low confluency and cultured in Endothelial cell medium (Cell 211-500). Fresh medium changed every other day and cells are split when close to confluency. For TNFα stimulation experiments, low passage cells were plated at 70% confluency and cultured overnight. Serum starvation was performed by changing cells to low-serum medium (0.5% FBS in basal endothelial medium (Lonza CC-3121)) for 2 hours. Human TNFα at concentration 10ng/mL was added to low-serum medium and incubated with cells for 90 minutes before cells were collected and assayed for downstream analysis. For experiments with Celastrol (Sigma C0869) or BRD4 inhibitors iBET151 (Cayman Chemical 11181) and UMN627 (provided by William Pomerantz, University of Minnesota), LSECs were plated and serum starved similarly to TNFα stimulation experiments. Inhibitors were added at concentrations indicated to low-serum medium, and cells were incubated in inhibitor containing medium for 2 hours. After

inhibitor treatment, medium change was performed with TNFα (Peprotech 300-01A) at 10ng/mL or control low-serum medium for 90 minutes incubation. LSECs were then collected for analysis.

Primary human monocytes (Astarte 1008) were thawed and resuspended in RPMI (Gibco 1640) with 10% bovine FBS supplemented with recombinant human M-CSF (BioLegend 574804) at 20ng/mL. Medium was changed on Day 1 then every 2 days and cells were cultured for up to 7 days.

CRISPR-dCas9 Lentiviral Transduction

Multiple sgRNA target sites were selected in the promoter area of *CXCL1* gene for dCas9-KRAB targeting assay. Putative target sites were selected based on predicted NF-κB binding motif analysis with JASPAR. The top five sites predicated to show the highest NF-κB binding affinity were chosen, and sgRNA sequence was designed using the publicly available Benchling software in proximity to these target sites. Synthesized sequences were inserted into the sgRNA backbone vector LentiGuide-Puro (Addgene Plasmid #52963) in accordance to protocol published by Feng et. al. The backbone vector was used as a control. The dCas9-KRAB Lentivector (Addgene Plasmid #89567) or a dCas9-FLAG Lentivector (Addgene Plasmid #106357) were also obtained through Addgene. 293T cells were cultured and transfected with either modified a sgRNA lentivector or a dCas9 lentivector according to manufacture protocol (Lipofectamine 3000, Invitrogen). Cells were cultured for 48 hours and supernatants containing lentivirus were collected. dCas9 lentivirus containing supernatant was further concentrated 100-fold with ultracentrifugation at 120,000g for 90 minutes (Optima XPN-80 Ultracentrifuge, Beckman Coulter). LSEC cells at low passage were transduced with supernatant containing sgRNA lentivirus along with 1:1000 dilution of polybrene (Millipore TR1003-G). Cells were cultured for 48hrs before selection with puromycin (puromycin resistance conferred by LentiGuide-Puro lentivector) (Sigma P8833). Selected cells were replated and transduced with either dCas9-KRAB or dCas9-FLAG lentivirus concentrate, and antibiotics selection was performed with either blasticidin (dCas9-KRAB) (Invitrogen ant-bl-1) or puromycin (dCas9-FLAG) on LSEC cells after 48 hours in culture. Selected cells were replated and treated with human TNFα and assayed by qPCR for CXCL gene expression.

CXCL1 ELISA

Human LSEC CXCL1 ELISA was performed on supernatant of cultured cells. Cultured cells underwent medium change with equal volume of culture medium for 16 hours before cells and supernatant media were collected. Capture ELISA was performed on supernatants using the Human CXCL1 DuoSet ELISA kit (R&D, DY275) using manufacturer's instructions. Fresh medium was used as negative control. Cells were collected and lysed in RIPA buffer (Cell Signaling 98065) and protein concentration was quantified with DC Protein Assay (BioRad 500-0114) according to manufacture protocols. CXCL1 concentrations determined by ELISA assays in supernatant was then normalized to protein concentration to ensure equal plating.

***In vitro* Cytotoxicity Studies**

LSECs or CRISPR dCas9-KRAB transformed cells were cultured in 96 well plates and imaged with IncuCyte (Essen Bioscience). For BET inhibitor toxicity study, cells were treated with varying levels of iBET151 along with TNFα at 10ng/mL. Incucyte® Cytotox Dye (Essen BioScience 4633)

was used to label dying cells and was added to the cells along with inhibitor iBET151 where appropriate. Cells were allowed to equilibrate at 37°C for 15 mins and transferred to IncuCyte for imaging at indicated time points at 10x magnification. 5 visual fields were captured per well. Quantification of the number or percentage of dead cells were done manually.

Immunohistochemistry

Right lobes of all mouse livers were fixed in 10% formalin and embedded in paraffin and cut into 5-μm sections. Slides were deparaffinized and underwent antigen retrieval (IHC-Tek IW-1000). Slides were treated with hydrogen peroxide for 10 minutes before blocking in 5% bovine serum albumin in phosphate-buffered saline (PBS) for 1 hour at room temperature. Samples were then incubated overnight at 4°C with rabbit anti-MPO antibody (Abcam ab9535) at dilution of 1:25. Slides were then blocked with Avidin and Biotin (Vector SP-2001) for 15 minutes each before treatment with biotinylated secondary antibody horse anti-Rabbit (Vector BA-1100) at 1:200 for 1 hour at room temperature. Slides were then treated with ABC reagent (Vector PK-7100) for 30 minutes before treatment with DAB reagent (Vector SK-4100) for 5 minutes. Counterstain was performed with hematoxylin stain and dehydrated in ethanol and xylene. Images were acquired using a Zeiss LSM 780 confocal microscope at 10x magnification, 5 images per slide (Carl Zeiss MicroImaging, Jena, Germany).

Oil Red Staining and BODIPY Staining

Liver sections from the left lobe of all mouse livers were embedded in OCT (Sakura 4583) and flash frozen. Frozen tissue was sectioned into 10μm slices, fixed in 10% formalin and stained with BODIPY 493/503 (Invitrogen D3922) or Oil-red (Sigma O0625) and counterstained with 4′,6-diamidino-2-phenylindole (DAPI) (Sigma D9542) or hematoxylin, respectively. Representative images were obtained with microscopy in one session under same settings (Carl Zeiss MicroImaging, Jena, Germany), 3 images per slide. The proportion of tissue stained with BODIPY, or Oil-red content was quantified with ImageJ macro commands for standardization. For BODIPY staining, FITC channel intensity was measured across the whole image, and average intensities of images were normalized to the average of all control samples. For Oil Red staining, red staining was quantified above a pre-selected threshold, and average intensities of images were normalized to the average of all control samples.

Western Blot

Liver tissue was homogenized in RIPA lysis buffer (Cell signaling 9806S) with protease inhibitor cocktail (Roche 4693159001). 40 ug of protein were loaded onto a SDS-PAGE gel for electrophoresis and proteins were transferred onto a nitrocellulose membrane. The membrane was blocked by 3% bovine serum albumin and then incubated overnight with a primary antibody. Primary antibodies used include: anti-caspase 3 (Cell signaling 14220S) at 1:1000 dilution and anti-HSC70 (Santa Cruz, sc7298) 1:5000 dilution. Blots were developed using a chemiluminescence substrate (Santa Cruz sc-2048). HSC70 was used as the loading control and the results were quantified by using the ImageJ software. Additional antibodies information in Supp Table 6.

Lipid Peroxidation Assay and 8-Hydroxy 2 Deoxyguanosine (8-OHdG) ELISA

MDA quantification with Lipid Peroxidation Assay Kit (Abcam ab118970) was performed according to manufacture protocol. For 8-OHdG assay, mice liver DNA was purified with DNeasy Blood&Tissue Kit (Qiagen 69504) and treated with Nuclease P1 according to manufacture protocol. 8-OHdG ELISA (Abcam ab201734) was performed according to manufacture protocol.

Supplementary Table 1: Summary of CXCLs Expression Data from Mouse and Human Liver

1.1 Bulk Liver RNA-seq Studies on *Cxcl1* Expression in Mouse Liver

	Treatment Conditions	Sources of *Cxcl1*	Isolation Strategy	References
1	Control vs. Conditional Kupffer Cell Depletion	↑HSC and LSEC, no change in Hepatocyte	Isolated Hepatocyte, LSEC, and HSC	GSE135790[31]
2	Control vs. TNFα Injection	↑LSEC, no change with Hepatocyte or Fibroblast	Isolated Hepatocyte, LSEC, and Fibroblast	GSE134663[32]

1.2 Single Cell RNA Sequencing Studies on *Cxcl* Expression in Mouse Liver (Gene Count per Cell Type as % of Total Gene Counts)

	Liver Condition	Top 3 Cell Types Producing Chemokines		Isolation Strategy	# Cells Sequenced	References
		Cxcl1	*Cxcl2*			
1	Normal	**LSEC 65%,** Macrophage 10%, Cholangiocyte 10%	Macrophage 62%, **LSEC 19%,** T cell 8%	NPC	17788	GSE148339[28]
2	Normal	Hepatocyte 75%, **LSEC 11%,** HSC 9%	Macrophage 95%	Whole liver lysate	10395	GSE129516[29]

1.3 Single Cell RNA Sequencing Studies on *CXCL* Expression in Human Liver (Gene Count per Cell Type as % of Total Gene Counts)

	**Liver Condition**	**Top 3 Cell Types Producing Chemokines**			**Isolation Strategy**	**# Cells Sequenced**	**References**
1	Normal	**CXCL1**	**CXCL2**	**CXCL3**	NPC	12264	GSE136103[27]
		Cholangiocyte 70%, **LSEC 8%** and Macrophage 7%	**LSEC 44%**, Cholangiocyte 27%, Macrophage 19%	Cholangiocyte 35%, Macrophage 32%, **LSEC 23%**			
		**CXCL5**	**CXCL6**	**CXCL8**			
		Cholangiocyte 49%, **LSEC 25%**, Macrophage 23%	Cholangiocyte 87%	Macrophage 56%, Cholangiocyte 21%, **LSEC 10%**			
	Cirrhotic	**CXCL1**	**CXCL2**	**CXCL3**	NPC	8962	
		Cholangiocyte 70%, **LSEC 16%**, Macrophage 6%	**LSEC 48%,** Macrophage 21%, Cholangiocyte 17%	Macrophage 39%, **LSEC 28%**, Cholangiocyte 23%			
		**CXCL5**	**CXCL6**	**CXCL8**			
		Cholangiocyte 90%	Cholangiocyte 88%	Macrophage 61%, Cholangiocyte 19%, **LSEC 9%**			
2	Normal	**CXCL1**	**CXCL2**	**CXCL3**	Cryopreserved/ fresh whole liver lysate	10327	GSE124395[26]
		Hepatocyte 69%, **LSEC 12%**, Cholangiocyte 9%	Gene Not Available in Dataset	Macrophage 42%, Cholangiocyte 24%, **LSEC 22%**			
		**CXCL5**	**CXCL6**	**CXCL8**			
		Cholangiocyte 82%, Hepatocyte 9%, **LSEC 5%**	Cholangiocyte 88%, Hepatocyte 6%	Macrophage 36%, Cholangiocyte 31%, **LSEC 18%**			
3	Normal	**CXCL1**	**CXCL2**	**CXCL3**	Parenchymal and NPC	20007	GSE115469[25]
		Cholangiocyte 50%, Macrophage 23%, **LSEC 11%**	Hepatocyte 67%, Macrophage 17%, **LSEC 12%**	Macrophage 74%, Cholangiocyte 14%, **LSEC 6%**			
		**CXCL5**	**CXCL6**	**CXCL8**			
		Cholangiocyte 86%, **LSEC 5%**	Cholangiocyte 85%	Macrophage 84%, Cholangiocyte 6%			

Supplemental Table 2: Mapping summary of RNA-seq libraries

Sample	Reads mapped to the genome (% of total)	Reads mapped to exon junction (% of total)	Total mapped reads (% of total)	Reads mapped to exons (% of total)	Total raw reads
Control 1	137068278 (80.1)	22868815 (13.4)	159937093 (93.4)	114398589 (66.8)	171184922
Control 2	130592832 (81.0)	20864454 (12.9)	151457286 (93.9)	108991415 (67.6)	161246524
Control 3	134135900 (81.2)	19882769 (12.0)	154018669 (93.3)	101846563 (61.7)	165157718
Control 4	115323919 (83.2)	9667289 (7.0)	124991208 (90.2)	56609355 (40.8)	138597116
AH1	146984642 (85.2)	15306695 (8.9)	162291337 (94.1)	95126412 (55.1)	172501600
AH2	115474148 (80.9)	17620215 (12.3)	133094363 (93.2)	93739851 (65.6)	142815554
AH3	112167894 (86.0)	11436381 (8.8)	123604275 (94.7)	68870657 (52.8)	130500686
AH4	101931947 (85.7)	8976303 (7.6)	110908250 (93.3)	59634507 (50.2)	118880144
AH5	141765494 (82.1)	17173420 (9.9)	158938914 (92.0)	97071388 (56.2)	172693632

Supplemental Table 3: Mapping summary of ChIP-seq and input libraries

Type	Mark	Both ends uniquely mapped (%)	One end uniquely mapped (%)	Both ends mapped to multiple locations (%)	Total pairs of reads
Control 1	H3K4me1	38127452 (80.4)	2583523 (5.45)	1663827 (3.51)	47423348
Control 2	H3K4me1	39602248 (81.56)	2838948 (5.85)	1837643 (3.78)	48553331
Control 3	H3K4me1	24559589 (77.83)	1770168 (5.61)	1192517 (3.78)	31554048
Control 4	H3K4me1	32109522 (81.34)	2309034 (5.85)	1458828 (3.7)	39473637
AH1	H3K4me1	26927752 (78.76)	2007086 (5.87)	1292433 (3.78)	34190367
AH2	H3K4me1	27479495 (80.47)	2036352 (5.96)	1433184 (4.2)	34148401
AH3	H3K4me1	29418252 (79.5)	1975280 (5.34)	1304917 (3.53)	37002707
AH4	H3K4me1	36586937 (67.55)	2260110 (4.17)	1686091 (3.11)	54159346
AH5	H3K4me1	29744254 (78.28)	2204911 (5.8)	1537798 (4.05)	37998643
Control 1	H3K4me3	27678021 (80.3)	1631633 (4.73)	1458590 (4.23)	34469219
Control 2	H3K4me3	31263188 (74.88)	2521722 (6.04)	2557658 (6.13)	41751385
Control 3	H3K4me3	21477751 (79.64)	1404722 (5.21)	1301506 (4.83)	26968401
AH1	H3K4me3	27385496 (74.58)	2150678 (5.86)	2122450 (5.78)	36721043
AH2	H3K4me3	26713750 (79.87)	1862844 (5.57)	1721281 (5.15)	33444624
AH3	H3K4me3	29264605 (74.63)	2273393 (5.8)	2172180 (5.54)	39212134
AH4	H3K4me3	17106615 (71.86)	1125946 (4.73)	1134855 (4.77)	23805439
AH5	H3K4me3	28131986 (77.54)	2577872 (7.1)	2362504 (6.51)	36282693
Control 1	H3K27ac	34395671 (82.28)	2053127 (4.91)	1241717 (2.97)	41803383
Control 2	H3K27ac	32693389 (80.43)	2133196 (5.25)	1325525 (3.26)	40648780
Control 3	H3K27Ac	20626118 (80.52)	1266871 (4.95)	798553 (3.12)	25615732
Control 4	H3K27ac	45703943 (80.58)	2850002 (5.03)	1773308 (3.13)	56715704
AH1	H3K27Ac	22516921 (69.73)	1195449 (3.7)	990814 (3.07)	32293862
AH2	H3K27ac	34894124 (79.52)	1937544 (4.42)	1440335 (3.28)	43880632
AH3	H3K27ac	31451725 (73.46)	1762226 (4.12)	1205388 (2.82)	42814409
AH4	H3K27Ac	40167419 (83.87)	2146946 (4.48)	1361800 (2.84)	47894755
AH5	H3K27ac	37578589 (84.58)	2026081 (4.56)	1302815 (2.93)	44431430
Control 1	H3K27me3	33617951 (75.09)	2751241 (6.14)	2365359 (5.28)	44772603
Control 2	H3K27me3	29073940 (75.26)	2529929 (6.55)	2381918 (6.17)	38629550
Control 3	H3K27me3	32346988 (75.5)	2844515 (6.64)	2537534 (5.92)	42844142
Control 4	H3K27me3	32741566 (74.21)	2723231 (6.17)	2602782 (5.9)	44121992
AH1	H3K27me3	27501675 (77.62)	2222310 (6.27)	1904928 (5.38)	35432547
AH2	H3K27me3	27970045 (77.6)	2281266 (6.33)	1930408 (5.36)	36045898
AH3	H3K27me3	28356788 (78.16)	2357069 (6.5)	1922705 (5.3)	36280699
AH4	H3K27me3	30955774 (75.83)	2674559 (6.55)	2171794 (5.32)	40824208
AH5	H3K27me3	32711607 (77.35)	2807348 (6.64)	2299261 (5.44)	42287779
Control 1	Input	29859878 (75.26)	2628374 (6.62)	2122976 (5.35)	39675619
Control 2	Input	26713181 (73.25)	2520310 (6.91)	2158943 (5.92)	36468614

Control 3	Input	41397842 (69.29)	3653318 (6.11)	3213332 (5.38)	59747797
Control 4	Input	32126876 (72.28)	2923344 (6.58)	2611351 (5.87)	44450694
AH1	Input	28461416 (73.27)	2602234 (6.7)	2301091 (5.92)	38844376
AH2	Input	28155775 (74.18)	2619247 (6.9)	2213286 (5.83)	37956273
AH3	Input	32441541 (76.17)	2842840 (6.68)	2401919 (5.64)	42588636
AH4	Input	11514874 (72.81)	1091049 (6.9)	859798 (5.44)	15815440
AH5	Input	26292584 (75.08)	2421047 (6.91)	2052937 (5.86)	35021604

Supplemental Table 4: qPCR Primers

Primer Table	
Primer Name	Sequence
Human CXCL1 Forward	GGAAAGCTTGCCTCAATCCT
Human CXCL1 Reverse	TTGTCACTGTTCAGCATCTTTTCG
Human CXCL2 Forward	CAGAAAGCTTGTCTCAACCCC
Human CXCL2 Reverse	TTTGCCATTTTTCAGCATCTTTTCG
Human CXCL6 Forward	ACGCTGAGAGTAAACCCCAA
Human CXCL6 Reverse	CCAGACAAACTTGCTTCCCG
Human CXCL8 Forward	CACTGCGCCAACACAGAAAT
Human CXCL8 Reverse	ACCCAGTTTTCCTTGGGGTC
Human GAPDH Forward	CCAGGGCTGCTTTTAACTCT
Human GAPDH Reverse	GGACTCCACGACGTACTCA
Human MTHFD2L Forward	CGGTGTGAGACATGAAGCCA
Human MTHFD2L Reverse	CCATGATTCCACACCTCGCT
Human RASSF6 Forward	ACAGGACCCAGATTCCTATGTC
Human RASSF6 Reverse	GCTGCTTCACTCATGGTTCTAT
Mouse CXCL1 Forward	CTGGGATTCACCTCAAGAACATC
Mouse CXCL1 Reverse	CAGGGTCAAGGCAAGCCTC
Mouse CXCL2 Forward	CCCTGCCAAGGGTTGACTTC
Mouse CXCL2 Reverse	GCAAACTTTTTGACCGCCCT
Mouse Ly6g Forward	GACTTCCTGCAACACAACTACC
Mouse Ly6g Reverse	ACAGCATTACCAGTGATCTCAGT
Mouse Beta-Actin Forward	CCTCCCTGGAGAAGAGCTATG
Mouse Beta-Actin Reverse	TTACGGATGTCAACGTCACAC
4C CXCL1 NlaIII Forward	GGCTCCGGGCTTTCCAG
4C CXCL1 Csp6I Reverse	CCTCTCTGCCTCAGCCTTTC
Illumina Adaptors LSEC Control	CAAGCAGAAGACGGCATACGAGATCGTGATGTGACTG GAGTTCAGACGTGTGCTCTTCCGATCTTTCAGTTTAGC CTATTTCACGTAC
Illumina Adaptors LSEC TNFa	CAAGCAGAAGACGGCATACGAGATATCACGGTGACTG GAGTTCAGACGTGTGCTCTTCCGATCTTTCAGTTTAGC CTATTTCACGTAC
Illumina Adaptors HEK293T	CAAGCAGAAGACGGCATACGAGATCGATGTGTGACTG GAGTTCAGACGTGTGCTCTTCCGATCTTTCAGTTTAGC CTATTTCACGTAC
3C NlaIII Enhancer Probe 1 F	GGAGAGAAGTTTCTGCAATTGTG
3C NlaIII CXCL1 Probe 1 R	CGGGATCGATCTGGAACTC
3C NlaIII CXCL2 Probe 1 R	CAGTTGTTGGGATTGAAAGTTAGG
3C NlaIII CXCL3 Probe 1 R	TATGTTAACCTCCCGGTCTCT
3C NlaIII CXCL5 Probe 1 R	ACTCAAGCTTTGGGATGCT
3C NlaIII CXCL6 Probe 1 R	CTGGGTCGTCAACCTTTGT
3C NlaIII CXCL8 Probe 1 R	GGAACCATCTCACTGTGTGTAA
3C NlaIII RASS Probe 1 R	GCTAAACCCAGGTACCATCAA
3C NlaIII Probe 1	TTTCCCTCACAGATGCCTCAAGAA
3C NlaIII Control A Probe 2-F	GAGAAGTTTCTGCAATTGTGACTTA
3C NlaIII Control A Probe 2-R	TCCTATTCATTTCCTTTCCCTTCT
3C NlaIII Control B Probe 2-F	GGAGAGAAGTTTCTGCAATTGTG
3C NlaIII Control B Probe 2-R	CCCAAAGTCCACGTACAAGT

3C NlaIII Control C Probe 2-F	TTCTGCAATTGTGACTTATCTACAG
3C NlaIII Control C Probe 2-R	AAATTAGCCAGGCGTGGT
3C NlaIII Control D Probe 2-F	TGGCAAAGTTAAGGAGAGAAG
3C NlaIII Control D Probe 2-R	TCGAGACCAGCCTAGCA
3C NlaIII Control E Probe 2-F	GGAGAGAAGTTTCTGCAATTGTG
3C NlaIII Control E Probe 2-R	ATCTCTCCTCCTGCACAGT
3C NlaIII Control F Probe 2-F	GTGGCTCCTAATGGCAAAGT
3C NlaIII Control F Probe 2-R	GATGCCCAGATTTATGATCCAGA
3C NlaIII Probe 2	TTCTTGAGGCATCTGTGAGGGAAA
ChIP NF-βB P1 F	CTCGGGATCGATCTGGAACT
ChIP NF-βB P1 R	TCTCCGAGATCCGCGAAC
ChIP NF-βB E1 F	TCCTTGGGAAATTCCACACAT
ChIP NF-βB E1 R	TCAGATTCCAGAGAGGTGACT
ChIP NF-βB P2 F	CTCCGAGAACGGCGAAC
ChIP NF-βB P2 R	GGCAGAAAGAGAACATCCCA
ChIP NF-βB P3 F	TATGCATGGTTGAGACTGGAAA
ChIP NF-βB P3 R	GAATATCCCAAAGTCCCAGAGTG
ChIP NF-βB P5 F	GTGCCTTCTGCACTCCTTT
ChIP NF-βB P5 R	AGGGTTACAACGTCCCTCT
ChIP NF-βB P6 F	CCCACCAATTCCCATCCTC
ChIP NF-βB P6 R	CATAGTGGTCAAGAGAGGGTTC
ChIP NF-βB P8 F	AAGATGAGGGTGCATAAGTTCTC
ChIP NF-βB P8 R	AGCTGCAGAAATCAGGAAGG
ChIP NF-βB E2 F	GCAATTCCAGCTGCTCATTC
ChIP NF-βB E2 R	GGGAGAATTTGGCACAAAGC
ChIP NF-βB E3 F	CAGATGAGTTCACCAGGCTATC
ChIP NF-βB E3 R	GTTGACTTGATCCTTCCCTCTT
ChIP NF-βB E4 F	GGTAAGTGAGGTCAGTGGAAAG
ChIP NF-βB E4 R	GTAAGTGCCTGAAGCACAATTTAT
ChIP H3K27ac P1 F	TCTCAATCTCCAGCCACAAATC
ChIP H3K27ac P1 R	GGACTCTGAGACTCTGGGATATT
ChIP H3K27ac E1 F	CCAGAAGGGAGACACAAGAAAT
ChIP H3K27ac E1 R	CCAGTGGGTTCTCAGACTTTAC
sgRNA- P1-Forward	CACCGTAAAAGGGGTTCGCGGATCT
sgRNA- P1-Reverse	AAACAGATCCGCGAACCCCTTTTA
sgRNA- CXCL SE1.6-Forward (E1)	CACCGTCCTTGGGAAATTCCACACA
sgRNA- CXCL SE1.6-Reverse (E1)	AAACTGTGTGGAATTTCCCAAGGAC
sgRNA- CXCL SE1.1-Forward	CACCGCACATGGTCAAGGGGCTCAT
sgRNA- CXCL SE1.1-Reverse	AAACATGAGCCCCTTGACCATGTGC
sgRNA- CXCL SE1.2-Forward	CACCGGGAAATTCCACACATGGTCA
sgRNA- CXCL SE1.2-Reverse	AAACTGACCATGTGTGGAATTTCCC
sgRNA- CXCL SE1.3-Forward	CACCGGAAATTCCACACATGGTCAA
sgRNA- CXCL SE1.3-Reverse	AAACTTGACCATGTGTGGAATTTCC
sgRNA- CXCL SE1.4-Forward	CACCGTGGGAAATTCCACACATGGT
sgRNA- CXCL SE1.4-Reverse	AAACACCATGTGTGGAATTTCCCAC
sgRNA- CXCL SE1.5-Forward	CACCGTCCTTGGGAAATTCCACACA
sgRNA- CXCL SE1.5-Reverse	AAACTGTGTGGAATTTCCCAAGGAC
sgRNA- CXCL SE2.1-Forward	CACCGAACAGGATGCCAGCACACTA

sgRNA- CXCL SE2.1-Reverse	AAACTAGTGTGCTGGCATCCTGTTC
sgRNA- CXCL SE2.2-Forward (E2)	CACCGTTCCTCTTTCATTGAATTGG
sgRNA- CXCL SE2.2-Reverse (E2)	AAACCCAATTCAATGAAAGAGGAAC
sgRNA- CXCL SE3.1-Forward (E3)	CACCGAGGAATTATCAAGATGTGAA
sgRNA- CXCL SE3.1-Reverse (E3)	AAACTTCACATCTTGATAATTCCTC
sgRNA- CXCL SE3.2-Forward	CACCGAAGATGTGAATGGAACATTC
sgRNA- CXCL SE3.2-Reverse	AAACGAATGTTCCATTCACATCTTC
sgRNA- CXCL SE3.3-Forward	CACCGTTCAGGCAGATGAGTTCACC
sgRNA- CXCL SE3.3-Reverse	AAACGGTGAACTCATCTGCCTGAAC
sgRNA- CXCL SE3.5-Forward	CACCGGGCTATCTAGAAACTAGGCG
sgRNA- CXCL SE3.5-Reverse	AAACCGCCTAGTTTCTAGATAGCCC
sgRNA- CXCL SE4.1-Forward	CACCGGGTGAGCATCATAGCCAATG
sgRNA- CXCL SE4.1-Reverse	AAACCATTGGCTATGATGCTCACCC
sgRNA- CXCL SE4.2-Forward (E4)	CACCGTGAGCATCATAGCCAATGTG
sgRNA- CXCL SE4.2-Reverse (E4)	AAACCACATTGGCTATGATGCTCAC
sgRNA- CXCL SE4.3-Forward	CACCGGAGCCACATTCTGCAGCTCT
sgRNA- CXCL SE4.3-Reverse	AAACAGAGCTGCAGAATGTGGCTCC

Supplemental Table 5: Accessed Public ChIP-seq Datasets in the Study

Data Set Name	Database Accession Number	Reference
HUVEC H3K27Ac ChIP-seq	GSE53998 (GSM1305204, GSM1305205)	[14]
HUVEC NF-κB ChIP-seq	GSE53998 (GSM1305210, GSM1305211)	[14]
HUVEC BRD4 ChIP-seq	GSE53998 (GSM1305201, GSM1305202)	[14]
HUVEC Hi-C	GSE63525 (GSM1551629, GSM1551630, GSM1551631)	[19]
LSEC Hi-C	ENCSR982KWR (ENCLB284TIY, ENCLB618NVM)	[21]
IMR90 Fibroblast Hi-C	GSE43070 (GSM1055800, GSM1055802)	[20]
Endothelial Precursor, Macrophage, Neutrophil Hi-C	EGAS00001001911	[23]
Mouse Macrophage NF-κB ChIP-seq	GSE16723 (GSM 11113, GSM11116)	[33]
Mouse Macrophage BRD4 ChIP-seq	GSE113226 (GSM3100539, GSM3100540)	[34]
Mouse Macrophage H3K27ac ChIP-seq	GSE31039 (GSM1000074)	[21]
Mouse Macrophage H3K4me1 ChIP-seq	GSE31039 (GSM1000066)	[21]
Mouse Macrophage H3K4me3 ChIP-seq	GSE31039 (GSM1000065)	[21]
Mouse Hepatocyte NF-kB ChIP-seq	GSE96767 (GSM2540285)	[35]
Mouse Hepatocyte H3K27ac ChIP-seq	GSE96767 (GSM2540257, GSM2540260)	[35]
Mouse Hepatocyte H3K4me3 ChIP-seq	GSE93431 (GSM2453285)	[36]
Mouse Liver Cell RNA-seq	GSE135790	[31]
Mouse Liver Cell RNA-seq	GSE134663	[32]
Mouse Liver scRNA-seq	GSE148339	[28]
Mouse Liver scRNA-seq	GSE129516	[29]
Human Liver scRNA-seq	GSE136103	[27]
Human Liver scRNA-seq	GSE124395	[26]
Human Liver scRNA-seq	GSE115469	[25]

Supplemental Table 6: Antibodies Used in the Study

Experiments	Antibodies	Primary/ Secondary	Supplier Name	Catalog Number	Clone Name	Lot Number	Validated Applications
Human Liver ChIP-seq/LSEC ChIP-seq	H3K27ac	Primary	Cell Signaling	8173	D5E4		Manufacture validated for WB, IF, IHC, Flow Cytometry, ChIP, ChIP-seq, Cut&Run
Human Liver ChIP-seq	H3K27me3	Primary	Cell Signaling	9733	C36B11		Manufacture validated for WB, IF, IHC, Flow Cytometry, ChIP, ChIP-seq, Cut&Run
Human Liver ChIP-seq	H3K4me1	Primary	Mayo In-House	EDL Lot 1			Previously validated and published[37]
Human Liver ChIP-seq	H3K4me3	Primary	Mayo In-House	EDL Lot 1			
LSEC ChIP-seq/LSEC ChIP-qPCR	NFkB	Primary	Cell Signaling	8242S	D14E12	13, 16	Manufacture validated for WB, IF, IHC, Flow Cytometry, ChIP, ChIP-seq, Cut&Run
LSEC ChIP-qPCR	BRD4	Primary	Abcam	ab128874	EPR5150 (2)	GR275920-39, GR3251918-5	Manufacture validated for WB, IHC-P, Flow-Cytometry, IF
LSEC ChIP-qPCR	H3K9me3	Primary	Abcam	ab8898		GR3302937-1	Manufacture validated for WB, IHC-P, ICC, ChIP
LSEC ChIP-qPCR	H3K27ac	Primary	Abcam	ab4729		GR3198866-1, GR3211959-1	Manufacture validated for ICC/IF, WB, IHC-P, ChIP, PepArr
LSEC ChIP-qPCR	Rabbit IgG Isotype control	Primary	Diagenode	C15410206		R2G001AG	Manufacture validated for ChIP
Mouse IHC	Myeloperoxidase	Primary	Abcam	ab9535		GR3194827-1, GR3304099-1, GR3273829-6	Manufacture validated for ICC, IHC-P
Caspase 3 WB	Caspase 3	Primary	Cell Signaling	14220s	D3R6Y	3	Manufacture validated for WB, IP
Caspase 3 WB	HSC70	Primary	Santa Cluz	sc7298		12520	Manufacture validated for WB, IP, IF, IHC(P), FCM and ELISA
Mouse IHC	Horse anti-Rabbit IgG Biotinolyated	Secondary	Vector	BA-1100		ZF1029, ZG0105	
Caspase 3 WB	Donkey anti-Rabbit IgG-HRP	Secondary	Amersham ECL	NA934V		17197685	
Caspase 3 WB	Sheep anti-Mouse IgG-HRP	Secondary	Amersham ECL	NA931V		17028693	

SUPPLEMENTAL REFERENCE

1 Kalari, K. R. *et al.* MAP-RSeq: Mayo Analysis Pipeline for RNA sequencing. *BMC Bioinformatics* **15**, 224, doi:10.1186/1471-2105-15-224 (2014).

2 Kim, D. *et al.* TopHat2: accurate alignment of transcriptomes in the presence of insertions, deletions and gene fusions. *Genome Biology* **14**, R36, doi:10.1186/gb-2013-14-4-r36 (2013).

3 Liao, Y., Smyth, G. K. & Shi, W. featureCounts: an efficient general purpose program for assigning sequence reads to genomic features. *Bioinformatics (Oxford, England)* **30**, 923-930, doi:10.1093/bioinformatics/btt656 (2014).

4 Zhong, J. *et al.* Purification of nanogram-range immunoprecipitated DNA in ChIP-seq application. *BMC Genomics* **18**, 985, doi:10.1186/s12864-017-4371-5 (2017).

5 Yan, H. *et al.* HiChIP: a high-throughput pipeline for integrative analysis of ChIP-Seq data. *BMC Bioinformatics* **15**, 280, doi:10.1186/1471-2105-15-280 (2014).

6 Li, H. & Durbin, R. Fast and accurate short read alignment with Burrows-Wheeler transform. *Bioinformatics (Oxford, England)* **25**, 1754-1760, doi:10.1093/bioinformatics/btp324 (2009).

7 Zhang, Y. *et al.* Model-based Analysis of ChIP-Seq (MACS). *Genome Biology* **9**, R137, doi:10.1186/gb-2008-9-9-r137 (2008).

8 Zang, C. *et al.* A clustering approach for identification of enriched domains from histone modification ChIP-Seq data. *Bioinformatics (Oxford, England)* **25**, 1952-1958, doi:10.1093/bioinformatics/btp340 (2009).

9 Quinlan, A. R. & Hall, I. M. BEDTools: a flexible suite of utilities for comparing genomic features. *Bioinformatics (Oxford, England)* **26**, 841-842, doi:10.1093/bioinformatics/btq033 (2010).

10 Shen, L., Shao, N., Liu, X. & Nestler, E. ngs.plot: Quick mining and visualization of next-generation sequencing data by integrating genomic databases. *BMC Genomics* **15**, 284, doi:10.1186/1471-2164-15-284 (2014).

11 Hamdan, F. H. & Johnsen, S. A. DeltaNp63-dependent super enhancers define molecular identity in pancreatic cancer by an interconnected transcription factor network. *Proceedings of the National Academy of Sciences* **115**, E12343-E12352, doi:10.1073/pnas.1812915116 (2018).

12 Tian, B., Nowak, D. E., Jamaluddin, M., Wang, S. & Brasier, A. R. Identification of direct genomic targets downstream of the nuclear factor-kappaB transcription factor mediating tumor necrosis factor signaling. *The Journal of biological chemistry* **280**, 17435-17448, doi:10.1074/jbc.M500437200 (2005).

13 Zhou, A., Scoggin, S., Gaynor, R. B. & Williams, N. S. Identification of NF-kappa B-regulated genes induced by TNFalpha utilizing expression profiling and RNA interference. *Oncogene* **22**, 2054-2064, doi:10.1038/sj.onc.1206262 (2003).

14 Brown, Jonathan D. *et al.* NF-κB Directs Dynamic Super Enhancer Formation in Inflammation and Atherogenesis. *Molecular Cell* **56**, 219-231 (2014).

15 Hah, N. *et al.* Inflammation-sensitive super enhancers form domains of coordinately regulated enhancer RNAs. *Proc Natl Acad Sci U S A* **112**, E297-302, doi:10.1073/pnas.1424028112 (2015).

16 Whyte, W. A. *et al.* Master transcription factors and mediator establish super-enhancers at key cell identity genes. *Cell* **153**, 307-319, doi:10.1016/j.cell.2013.03.035 (2013).

17 Eisenberg, E. & Levanon, E. Y. Human housekeeping genes, revisited. *Trends in genetics : TIG* **29**, 569-574, doi:10.1016/j.tig.2013.05.010 (2013).

18 Yang, D. *et al.* 3DIV: A 3D-genome Interaction Viewer and database. *Nucleic Acids Research* **46**, D52-D57, doi:10.1093/nar/gkx1017 (2017).

19 Rao, S. S. *et al.* A 3D map of the human genome at kilobase resolution reveals principles of chromatin looping. *Cell* **159**, 1665-1680, doi:10.1016/j.cell.2014.11.021 (2014).
20 Jin, F. *et al.* A high-resolution map of the three-dimensional chromatin interactome in human cells. *Nature* **503**, 290-294, doi:10.1038/nature12644 (2013).
21 Consortium, E. P. An integrated encyclopedia of DNA elements in the human genome. *Nature* **489**, 57-74, doi:10.1038/nature11247 (2012).
22 Schofield, E. C. *et al.* CHiCP: a web-based tool for the integrative and interactive visualization of promoter capture Hi-C datasets. *Bioinformatics (Oxford, England)* **32**, 2511-2513, doi:10.1093/bioinformatics/btw173 (2016).
23 Javierre, B. M. *et al.* Lineage-Specific Genome Architecture Links Enhancers and Non-coding Disease Variants to Target Gene Promoters. *Cell* **167**, 1369-1384.e1319 (2016).
24 Hao, Y. *et al.* Integrated analysis of multimodal single-cell data. *bioRxiv*, 2020.2010.2012.335331, doi:10.1101/2020.10.12.335331 (2020).
25 MacParland, S. A. *et al.* Single cell RNA sequencing of human liver reveals distinct intrahepatic macrophage populations. *Nat Commun* **9**, 4383, doi:10.1038/s41467-018-06318-7 (2018).
26 Aizarani, N. *et al.* A human liver cell atlas reveals heterogeneity and epithelial progenitors. *Nature* **572**, 199-204, doi:10.1038/s41586-019-1373-2 (2019).
27 Ramachandran, P. *et al.* Resolving the fibrotic niche of human liver cirrhosis at single-cell level. *Nature* **575**, 512-518, doi:10.1038/s41586-019-1631-3 (2019).
28 Xiong, X. *et al.* Landscape of Intercellular Crosstalk in Healthy and NASH Liver Revealed by Single-Cell Secretome Gene Analysis. *Mol Cell* **75**, 644-660 e645, doi:10.1016/j.molcel.2019.07.028 (2019).
29 Nault, R., Fader, K. A., Bhattacharya, S. & Zacharewski, T. R. Single-Nuclei RNA Sequencing Assessment of the Hepatic Effects of 2,3,7,8-Tetrachlorodibenzo-p-dioxin. *Cell Mol Gastroenterol Hepatol* **11**, 147-159, doi:10.1016/j.jcmgh.2020.07.012 (2021).
30 Walter, C., Schuetzmann, D., Rosenbauer, F. & Dugas, M. Basic4Cseq: an R/Bioconductor package for analyzing 4C-seq data. *Bioinformatics (Oxford, England)* **30**, 3268-3269, doi:10.1093/bioinformatics/btu497 (2014).
31 Bonnardel, J. *et al.* Stellate Cells, Hepatocytes, and Endothelial Cells Imprint the Kupffer Cell Identity on Monocytes Colonizing the Liver Macrophage Niche. *Immunity* **51**, 638-654 e639, doi:10.1016/j.immuni.2019.08.017 (2019).
32 Krausgruber, T. *et al.* Structural cells are key regulators of organ-specific immune responses. *Nature* **583**, 296-302, doi:10.1038/s41586-020-2424-4 (2020).
33 Barish, G. D. *et al.* Bcl-6 and NF-kappaB cistromes mediate opposing regulation of the innate immune response. *Genes & development* **24**, 2760-2765, doi:10.1101/gad.1998010 (2010).
34 Dey, A. *et al.* BRD4 directs hematopoietic stem cell development and modulates macrophage inflammatory responses. *EMBO J* **38**, doi:10.15252/embj.2018100293 (2019).
35 Goldstein, I., Paakinaho, V., Baek, S., Sung, M. H. & Hager, G. L. Synergistic gene expression during the acute phase response is characterized by transcription factor assisted loading. *Nat Commun* **8**, 1849, doi:10.1038/s41467-017-02055-5 (2017).
36 Schwarzer, W. *et al.* Two independent modes of chromatin organization revealed by cohesin removal. *Nature* **551**, 51-56, doi:10.1038/nature24281 (2017).
37 Argemi, J. *et al.* Defective HNF4alpha-dependent gene expression as a driver of hepatocellular failure in alcoholic hepatitis. *Nature Communications* **10**, 3126, doi:10.1038/s41467-019-11004-3 (2019).
