# Supplementary material for: Super enhancer regulation of cytokine-induced chemokine production in alcoholic hepatitis
Source: Nat Commun. 2021 Jul 27;12:4560. doi: 10.1038/s41467-021-24843-w (PMC8316465; doi:10.1038/s41467-021-24843-w)
Supplement: Supplementary file 3 — Reporting Summary [file 41467_2021_24843_MOESM3_ESM.pdf]

nature research

Corresponding author(s): Vijay H. Shah and Huihuang Yan

Last updated by author(s): June 5, 2021

Reporting Summary

Nature Research wishes to improve the reproducibility of the work that we publish. This form provides structure for consistency and transparency in reporting. For further information on Nature Research policies, see our Editorial Policies and the Editorial Policy Checklist.

Statistics

For all statistical analyses, confirm that the following items are present in the figure legend, table legend, main text, or Methods section.

n/a	Confirmed	
☐	☒	The exact sample size (n) for each experimental group/condition, given as a discrete number and unit of measurement
☐	☒	A statement on whether measurements were taken from distinct samples or whether the same sample was measured repeatedly
☐	☒	The statistical test(s) used AND whether they are one- or two-sided<br>*Only common tests should be described solely by name; describe more complex techniques in the Methods section.*
☒	☐	A description of all covariates tested
☐	☒	A description of any assumptions or corrections, such as tests of normality and adjustment for multiple comparisons
☐	☒	A full description of the statistical parameters including central tendency (e.g. means) or other basic estimates (e.g. regression coefficient) AND variation (e.g. standard deviation) or associated estimates of uncertainty (e.g. confidence intervals)
☐	☒	For null hypothesis testing, the test statistic (e.g. F, t, r) with confidence intervals, effect sizes, degrees of freedom and P value noted<br>*Give P values as exact values whenever suitable.*
☒	☐	For Bayesian analysis, information on the choice of priors and Markov chain Monte Carlo settings
☒	☐	For hierarchical and complex designs, identification of the appropriate level for tests and full reporting of outcomes
☒	☐	Estimates of effect sizes (e.g. Cohen's d, Pearson's r), indicating how they were calculated

Our web collection on statistics for biologists contains articles on many of the points above.

Software and code

Policy information about availability of computer code

Data collection	No software was used for data collection.
Data analysis	Basic4Cseq v1.0.2; BEDTools v2.16.2; BWA v0.5.9; BWA-MEM v0.7.10; DiffBind v2.4.8; edgeR v3.18.1; featureCounts v1.4.6; igvtools v2.2.2; MACS2 v2.0.10; ngs.plot v2.02; SICER v1.1; Picard v1.67; ROSE v1.0.0; TopHat v2.1.0.; GSEA v4.0.0; PRISM 8

For manuscripts utilizing custom algorithms or software that are central to the research but not yet described in published literature, software must be made available to editors and reviewers. We strongly encourage code deposition in a community repository (e.g. GitHub). See the Nature Research guidelines for submitting code & software for further information.

Data

Policy information about availability of data

All manuscripts must include a data availability statement. This statement should provide the following information, where applicable:
- Accession codes, unique identifiers, or web links for publicly available datasets
- A list of figures that have associated raw data
- A description of any restrictions on data availability

-Figure 1 utilized liver RNA-seq/ChIP-seq data (GSE155926)
-Figure 3b utilized liver ChIP-seq data (GSE155926), Figure 3d-e LSEC ChIP-seq data (GSE166564)
-Supp Fig 2 utilized liver RNA-seq data (GSE155926)
-Supp Fig 3 utilized liver RNA-seq/ChIP-seq data (GSE155926)
-Supp Fig 4 utilized liver RNA-seq/ChIP-seq data (GSE155926)
-Supp Fig 5 utilized liver RNA-seq/ChIP-seq data (GSE155926)
-Supp Fig 11 utilized LSEC ChIP-seq data (GSE166564)

-No restrictions on data availability
-JASPAR database accessed from http://jaspar.genereg.net/

Field-specific reporting

Please select the one below that is the best fit for your research. If you are not sure, read the appropriate sections before making your selection.

☒ Life sciences ☐ Behavioural & social sciences ☐ Ecological, evolutionary & environmental sciences

For a reference copy of the document with all sections, see nature.com/documents/nr-reporting-summary-flat.pdf

Life sciences study design

All studies must disclose on these points even when the disclosure is negative.

Sample size	4 healthy and 5 AH individuals were used in ChIP-seq and 4 healthy and 6 AH individuals were included in bulk RNA-seq. No sample size calculation was performed. Sample size was sufficient for this exploratory assessment of AH gene expression and histone modification.
Data exclusions	No data were excluded from the study.
Replication	We generated genome-wide maps for 4 histone modifications including H3K4me1, H3K4me3, H3K27ac, and H3K27me3 in 4 healthy and 5 AH individuals. We generated RNA-seq data in 4 healthy and 6 AH individuals. The different subjects within healthy and AH groups were treated as replicates, respectively, in differential binding analysis and differential gene expression analysis between the two groups. Whole genome sequencing data was not performed without technical replicates but quality control assessment was performed as described. Low quality samples were not used in subsequent analysis.
Randomization	Randomization is not applicable given exploratory nature of the experiment.
Blinding	Blinding is not applicable given exploratory nature of the experiment.

Reporting for specific materials, systems and methods

We require information from authors about some types of materials, experimental systems and methods used in many studies. Here, indicate whether each material, system or method listed is relevant to your study. If you are not sure if a list item applies to your research, read the appropriate section before selecting a response.

Materials & experimental systems

n/a	Involved in the study	
☐	☒	Antibodies
☐	☒	Eukaryotic cell lines
☒	☐	Palaeontology and archaeology
☐	☒	Animals and other organisms
☐	☒	Human research participants
☒	☐	Clinical data
☒	☐	Dual use research of concern

Methods

n/a	Involved in the study	
☐	☒	ChIP-seq
☒	☐	Flow cytometry
☒	☐	MRI-based neuroimaging

Antibodies

Antibodies used	A table summarizing antibody information is added to Supplemental Information, Supplementary Table 6.
Validation	All antibodies used were validated for their use in perspective experiments, based on the company website. This information is provided in Supplemental Table 6.

Eukaryotic cell lines

Policy information about cell lines

Cell line source(s)	HEK293T and HepG2: ATCC:CRL-11268; ATCC Cat: HB-8065; Primary cells LSEC, HSC and HBiEC are from ScienCell Research Laboratories Cat:5000, Cat:5300 and Cat:5100
Authentication	Cell lines were not authenticated
Mycoplasma contamination	Cell lines were tested for mycoplasma every 6 months with the PlasmoTest™ - Mycoplasma Detection Kit from Invivogen, and results has been negative.

Commonly misidentified lines (See ICLAC register)	None were used in the study

Animals and other organisms

Policy information about studies involving animals; ARRIVE guidelines recommended for reporting animal research

Laboratory animals	Wildtype C57BL/6 mice from Envigo Laboratories were used in the study. Mice were aged 10-12 weeks. All mice used in experiment were female to minimize heterogeneity of alcohol feeding. Mice were kept in controlled environment, with 12 hours of light/dark (6PM-6AM), ambient temperature at 68-79°F (~20-26°C) with 30-70% humidity.
Wild animals	No wild animals were used.
Field-collected samples	Study did not involve any samples collected from the field.
Ethics oversight	All animal work was performed under Mayo Institutional Animal Care and Use Committee oversight, in AAALAC-accredited facilities.

Note that full information on the approval of the study protocol must also be provided in the manuscript.

Human research participants

Policy information about studies involving human research participants

Population characteristics	Liver samples were obtained from patients with alcoholic hepatitis at the time of liver transplant or patients with no known chronic liver disease who underwent liver resection for another cause (such as resection of benign liver lesions). Both male and female subjects were included in both groups, age range for patients with AH was 32-60, and control was 58-71. Patients in the AH group were diagnosed with severe AH, and patients in control groups had no known liver disease. Further clinical characteristics are summarized in Supp Fig 1.
Recruitment	Alcoholic hepatitis patients were recruited around the time of liver transplant for permission to use explant liver tissue for research studies at University of Lille, France. Control patients were recruited around the time of liver resection surgery for permission to use resected liver tissue for research studies at Mayo Clinic, Rochester, MN. No expect biases, including self-selection bias was expected from patient recruitment. Recruitment and participation in the trial has no impact on patient's clinical treatment course or prognosis.
Ethics oversight	IRB protocols were approved by the Ethics Committee of the University of Lille, France for alcoholic hepatitis samples, and Mayo Clinic for control patients. Studies were performed with informed consent and in accordance with the Declaration of Helsinki.

Note that full information on the approval of the study protocol must also be provided in the manuscript.

ChIP-seq

Data deposition

- [x] Confirm that both raw and final processed data have been deposited in a public database such as GEO.
- [x] Confirm that you have deposited or provided access to graph files (e.g. BED files) for the called peaks.

Data access links *May remain private before publication.*	All RNA-seq and ChIP-seq data generated in this publication will be available publication of this manuscript on the GEO database (GSE155926 and166564).
Files in database submission	All RNA-Seq and ChIP-Seq files used in this study
Genome browser session (e.g. UCSC)	No longer applicable

Methodology

Replicates	4 normal and 5 AH samples were included in ChIP-seq.
Sequencing depth	51 bp paired-end reads. Between 23.8 and 56.7 million pairs of raw reads were generated per sample.
Antibodies	anti-H3K27ac antibody (CST, #8173), in-house generated anti-H3K4me3 antibody (EDL lot 1), in-house generated anti-H3K4me1 antibody (EDL lot 1), and anti-H3K27me3 antibody (CST, #9733)
Peak calling parameters	Narrow peaks were identified with MACS2 v2.0.10 with the parameter settings "-f BAM -g hs --keep-dup all -q 0.01 –nomodel --shiftsize=half_of_median_fragment-size". Broad peaks (for H3K27me3) were identified using SICER v1.1 with the parameter settings "hg19 1 200 300 0.75 600 1E-2".
Data quality	ChIP-seq data quality was assessed according to the ENCODE quality standards (https://www.encodeproject.org/chip-seq/histone/).
Software	Raw reads were mapped to the hg19 genome reference using Burrows-Wheeler Aligner (BWA v0.5.9). Pairs of reads with one or both ends uniquely mapped were retained. Alignments were position sorted using the Picard SortSam command and duplicates were

removed using Picard MarkDuplicates command (http://broadinstitute.github.io/picard/, picard-tools v1.67). H3K4me1, H3K4me3 and H3K27ac peaks were identified using MACS2 (v2.0.10) at the parameter setting “-f BAM -g hs --keep-dup all -q 0.01 –nomodel --shiftsize=half_of_median_fragment-size”. H3K27me3 peaks were identified using SICER (v1.1) at the parameter setting "hg19 1 200 300 0.75 600 1E-2".
